# Sustaining Compassionate Care in Pediatric and Neonatal Palliative Care: A Qualitative Meta‐Synthesis of Nurses’ Experiences Amid Uncertainty and Loss

**DOI:** 10.1155/jonm/5143378

**Published:** 2026-11-01

**Authors:** Yuan Li, Shiqi Luo, Xia Li, Hanmei Peng, Yanling Hu, Qiong Chen

**Affiliations:** ^1^ Department of Neonatology Nursing West China Second University Hospital Sichuan University Chengdu 610041 China scu.edu.cn; ^2^ Key Laboratory of Birth Defects and Related Diseases of Women and Children Ministry of Education Sichuan University Chengdu 610041 China scu.edu.cn; ^3^ Department of Pediatrics West China Second University Hospital Sichuan University Chengdu 610041 China scu.edu.cn

**Keywords:** coping, meaning-making, neonatal palliative care, nurses, pediatric palliative care, professional boundaries, qualitative meta-synthesis

## Abstract

**Background:**

Nurses provide pediatric and neonatal palliative care amid substantial emotional and relational demands. Qualitative evidence remains fragmented on how nurses sustain compassionate care and how relational and organizational conditions shape these efforts. This systematic review and meta‐synthesis examined these experiences across palliative and end‐of‐life settings.

**Methods:**

PubMed, CINAHL, Embase, PsycINFO, Web of Science, and Scopus were searched from inception to September 2026. Qualitative studies and separately extractable qualitative components of mixed‐methods studies addressing nurses’ experiences of pediatric or neonatal palliative care were eligible. Two reviewers independently screened records, extracted data, and applied the Joanna Briggs Institute qualitative appraisal checklist. Findings were synthesized using Thomas and Harden’s three‐stage thematic synthesis, with confidence assessed using GRADE‐CERQual.

**Results:**

Thirty‐seven studies from 21 countries reported a total of 599 nursing participants. Four analytical themes and 18 descriptive subthemes were generated: (1) finding meaning through care for children and families; (2) managing relational closeness and professional boundaries; (3) responding to grief and loss through coping and meaning‐making; and (4) seeking and using interpersonal and organizational support. Confidence was moderate for Themes 1–3 and high for Theme 4. Valued care, relational involvement, coping, and support coexisted with persistent grief, emotional depletion, and unmet needs.

**Conclusions:**

Nurses’ efforts to sustain compassionate care involve interacting personal, relational, and organizational processes. Meaningful care can coexist with persistent grief and emotional strain, with variation in emotional availability and recovery. These findings highlight the importance of organizational support alongside individual coping.

**Implications for Nursing Management:**

Managers should protect time and resources for relational care and strengthen nursing participation in interdisciplinary decisions. Emotional and clinical support should be accessible and responsive to nurses’ preferences, including opportunities for reflection, supervision, and peer support.

## 1. Introduction

An estimated 21 million children worldwide could benefit from palliative care each year, of whom more than 8 million may require specialized pediatric palliative care [1, 2]. Pediatric and neonatal palliative care provides active, holistic, and family‐centered support for newborns through adolescents with life‐limiting or life‐threatening conditions across hospital, hospice, community, and home settings [3, 4]. Developmental differences shape children’s capacity to communicate, understand illness, and participate in decisions, with parents often assuming a central role as surrogate decision‐makers [5, 6]. Palliative care may begin at diagnosis alongside disease‐directed treatment, with the emphasis shifting as prognosis and treatment goals evolve [7]. In neonatal settings, decisions about life‐sustaining treatment may arise before or soon after birth under substantial prognostic uncertainty [8]. Across age groups, nurse–family therapeutic relationships may extend from diagnosis into bereavement. Deterioration, death, and bereavement place substantial emotional, relational, and clinical demands on clinicians, who must address suffering, uncertainty, and family needs [5, 9].

Nurses occupy a distinctive position within this relationally intensive care. Through frequent direct contact with children and families, nurses can recognize subtle clinical changes, provide symptom relief and comfort, and communicate clinical information [3]. They also advocate for children’s and families’ preferences and accompany families before and after death [3]. These activities support compassionate care through family‐centered responses to suffering that preserve dignity and address children’s and families’ needs [10, 11]. This sustained bedside presence, combining physical proximity with relational attentiveness, may reinforce professional meaning while increasing emotional vulnerability as relationships deepen and losses recur [12–14]. Providing the care involves emotional labor, described by Smith and Badolamenti et al. as regulating nurses’ own emotions and expressions while attending to patients’ emotional needs [15–17]. The Emotional Labor Model in Pediatric Nursing connects this work to the child and family, the nurse, and their therapeutic relationship [16].

Research in pediatric palliative care highlights education, reflection, and organizational support for managing these emotional demands and addressing burnout risk [18]. To cope with difficult encounters, nurses manage professional boundaries and draw on emotional processing, self‐care, collegial support, spiritual or cultural resources, and strategic distancing [19, 20]. Insufficient preparation and fragmented support may undermine these efforts and compound distress after loss [19, 21, 22]. Responsive leadership, adequate staffing and resources, interprofessional collaboration, and nursing participation in decision‐making may support staff well‐being and professional engagement [23, 24]. Sustaining compassionate care is therefore a nursing management responsibility, with implications for workforce sustainability.

Existing qualitative studies and reviews have advanced understanding of nurses’ grief, occupational stress, moral distress, coping, and relational challenges in pediatric and neonatal palliative care [25–27]. However, the evidence remains dispersed across populations, settings, and dimensions of nursing experience. This systematic review and meta‐synthesis aimed to identify, appraise, and synthesize qualitative evidence on nurses’ experiences of sustaining compassionate care in pediatric and neonatal palliative and end‐of‐life settings, and sought to interpret how personal, relational, and organizational processes enable or constrain nurses’ therapeutic and emotional availability.

## 2. Methods

### 2.1. Study Design

This study employed a systematic review and qualitative meta‐synthesis design. The review protocol was prospectively registered with PROSPERO (CRD420261441995). Review procedures were guided by the Joanna Briggs Institute (JBI) methodology for qualitative evidence synthesis [28]. Reporting followed the Enhancing Transparency in Reporting the Synthesis of Qualitative Research (ENTREQ) statement [29] and the Preferred Reporting Items for Systematic Reviews and Meta‐Analyses (PRISMA) 2020 statement [30].

### 2.2. Search Strategy

PubMed, CINAHL, Embase, PsycINFO, Web of Science, and Scopus were searched from database inception to September 2026, with no restrictions on language or publication year. Searches combined four concept blocks: nursing, pediatric/neonatal populations, palliative/end‐of‐life care, and qualitative research. Controlled vocabulary and free‐text terms were adapted to each database, as detailed in Supporting Material A. Reference lists of included studies and relevant reviews were also screened. The exact database‐specific fields and strings, including the more restricted palliative‐care block in Embase, are reported in Supporting Material A.

### 2.3. Inclusion and Exclusion Criteria

The review question and a priori eligibility criteria were structured using the framework Population, Phenomenon of Interest, and Context (PICo) for qualitative evidence synthesis [31]. PICo was appropriate because the review focused on nurses’ experiences and the factors shaping their ability to sustain compassionate care within specific care contexts. Studies were eligible if they met all of the following criteria: (1) Population: registered nurses or equivalent licensed nursing professionals; (2) Phenomenon of Interest: findings addressing how nurses experienced, managed, or responded to the emotional, relational, or professional demands of pediatric or neonatal palliative and end‐of‐life care, including factors that enabled or constrained their ability to continue providing care; (3) context: hospital, hospice, or community settings involving neonates, infants, children, or adolescents aged 0–18 years, including care during dying and the immediate postdeath period; and (4) study design: qualitative studies, or mixed‐methods studies with separately identifiable qualitative findings, published as full‐text articles in peer‐reviewed journals. Studies involving mixed professional groups or mixed age populations were included only when nurse‐specific findings and pediatric or neonatal data could be clearly extracted. Commentaries, editorials, reviews, protocols, quantitative‐only studies, and reports without extractable qualitative findings relevant to the review question were excluded.

### 2.4. Study Selection and Data Extraction

Retrieved records were imported into EndNote X9 for reference management and duplicate removal. Two reviewers (LY and LSQ) independently screened titles and abstracts and subsequently assessed the full texts of potentially eligible reports. Discrepancies were resolved through discussion, with consultation from a third senior reviewer (CQ) when consensus could not be reached. Two reviewers independently extracted data using a standardized form piloted on representative studies. Extracted information included bibliographic details, country, study setting, participant characteristics, study design, sampling and recruitment methods, data collection and analysis methods, and findings relevant to the review question. All eligible companion reports were retained and linked to the same underlying study, with their findings integrated under a single study identifier.

### 2.5. Quality Appraisal

The methodological quality of the included studies was independently assessed by two reviewers using the JBI Critical Appraisal Checklist for Qualitative Research [28]. The 10‐item checklist assesses congruity between the philosophical perspective, methodology, research question, data collection, analysis, and interpretation. It also considers researcher positioning and influence, participants’ voices, ethical conduct, and whether conclusions follow from the analysis. Each criterion was rated as “Yes,” “No,” or “Unclear” in accordance with JBI guidance. Disagreements were resolved through discussion, with consultation from a third reviewer when consensus could not be achieved.

### 2.6. Data Synthesis

Qualitative findings were synthesized using Thomas and Harden’s three‐stage thematic synthesis [32]. First, two reviewers (LY and LSQ) independently conducted line‐by‐line coding of the extracted findings using Microsoft Excel, generating initial codes that remained close to the language and meanings of the primary studies. Second, related initial codes were compared within and across studies and grouped into descriptive subthemes representing recurring patterns in nurses’ experiences. Third, descriptive subthemes were compared across settings and participant groups to develop analytical themes addressing the review question. Candidate themes were iteratively checked against the extracted findings and supporting quotations for conceptual fit, coherence, and distinctiveness. An audit trail linking primary‐study findings to initial codes, descriptive subthemes, and analytical themes was maintained throughout the synthesis. Coding decisions and theme labels were discussed within the review team until consensus was reached.

### 2.7. Confidence Assessment

Confidence in each synthesized finding was evaluated using GRADE‐CERQual [33]. Four components were considered: Methodological limitations, coherence, adequacy of data, and relevance to the review question. Methodological limitations were informed by the study‐level JBI appraisal; coherence reflected the extent to which findings across contributing studies formed a consistent pattern; adequacy considered the richness and quantity of supporting data; and relevance assessed the extent to which the contributing studies aligned with the review’s PICo criteria. Overall confidence was rated as high, moderate, low, or very low based on concerns identified across the four components. Reasons for component judgments and overall ratings are reported in Supporting Table 3.

## 3. Results

### 3.1. Search Results

Database searches identified 3847 records. After 1800 duplicates were removed, 2047 titles and abstracts were screened, and 140 reports underwent full‐text assessment. Reference‐list screening identified 39 additional reports. Following full‐text exclusions (Supporting Table 1), 39 reports representing 37 studies were included in the meta‐synthesis (Figure 1).

**FIGURE 1:**
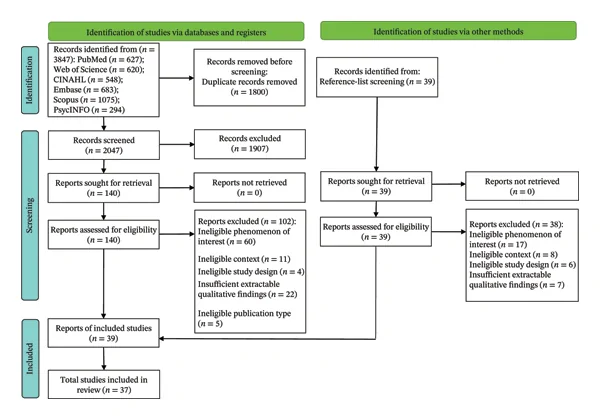
PRISMA flow diagram of the study selection process.

### 3.2. Characteristics of the Included Studies

The 37 included studies were published between 1999 and 2026 and involved 599 nursing participants across 21 countries on six continents; the United States contributed the most studies (*n* = 7). Studies were conducted across hospital, hospice, community, and home settings, including NICUs, PICUs, and pediatric oncology and general pediatric units. Participants were primarily registered nurses; some studies also included nurse practitioners or other healthcare professionals, from whom only nurse‐specific findings were extracted. Phenomenological and qualitative descriptive designs predominated, with grounded theory, narrative, and other interpretive approaches, and mixed‐methods designs also represented. Summary characteristics are presented in Table 1, with full study details provided in Supporting Table 2.

**TABLE 1:** Summary characteristics of the included studies (*n* = 37).

Authors, year, country	Study design	Setting	Participants (*n*)	Data collection	Data analysis
Abbasi, 2026, Iran [19]	Descriptive qualitative study	Pediatric oncology department	20 nurses	Unstructured and in‐depth semistructured interviews	Content analysis
Alm, 2026, Denmark [12]	Phenomenological‐hermeneutical study	Regional home‐based pediatric palliative care	10 nurses	Individual interviews	Thematic reflection
Anguis‐Carreño, 2023, Spain [34]	Hermeneutic phenomenology study	PICU	10 nurses	Semistructured individual interviews	Latent content analysis
Bassola, 2023, Italy [35]	Qualitative, phenomenological descriptive study	Pediatric ward and NICU	27 nurses	In‐depth interviews	Phenomenological analysis
Bian, 2023, China [36]	Descriptive qualitative study	Pediatric ward	10 nurses	Semistructured interviews	Content analysis
Bloomer, 2015, Australia [37]	Mixed‐methods study	NICU and PICU	22 nurses	Focus groups and individual interviews	Content analysis
Briere, 2025, USA [38]	Qualitative descriptive study	NICU and PICU	20 nurses/nurse practitioners	Zoom interviews	Qualitative thematic analysis
Brimble, 2024, UK [39]	Narrative interpretive approach study	Children’s hospice respite/day care	6 nurses	Audio diaries followed by telephone interviews	Inductive thematic analysis
Budak, 2025, Turkey [40]	Phenomenological study	Pediatric units	18 nurses	In‐depth semistructured interviews	Thematic analysis
Chew, 2021, Singapore [41]	Descriptive qualitative study	Pediatric setting	12 nurses	Semistructured interviews	Thematic analysis
Chong, 2017, Malaysia [21]	Qualitative study	Community palliative‐care provider organizations	16 nurses	Semistructured in‐depth interviews	Thematic analysis
Cook, 2012, USA [42]	Qualitative descriptive study	Acute cardiology unit	22 nurses/nurse practitioners	Focus groups	Content analysis
Devitt, 2021, Ireland [43]	Qualitative study	Pediatric wards	14 nurses	Semistructured interviews	Thematic analysis
Ebadinejad, 2022, Iran [44]	Conventional qualitative study	Pediatric cancer ward	16 participants including 8 nurses	In‐depth individual unstructured and semistructured interviews	Content analysis
Elsharkawy, 2025, Saudi Arabia [45]	Hermeneutic phenomenological study	NICU	18 nurses	Semistructured interviews, reflective journals and follow‐up interviews	Interpretive phenomenological analysis
Epstein, 2008 and 2010, USA [46, 47]	Hermeneutic phenomenological qualitative study	NICU	21 nurses and 11 physicians	Semistructured interviews	Hermeneutic circle analysis; conventional content analysis
Erikson, 2017, USA [48]	Constructivist grounded theory study	PPC center, oncology unit, intensive care nursery and PICU	18 registered nurses	Semistructured interviews	Constructivist grounded theory analysis
Gürcan, 2025, Turkey [49]	Grounded theory study	Pediatric hematology and oncology clinics	14 nurses	Face‐to‐face and online semistructured interviews with researcher field notes	Grounded theory analysis with constant comparative analysis
Henao‐Castaño, 2019, Colombia [50]	Hermeneutic phenomenological study	PICU	10 nurses	Semistructured in‐depth interviews	Phenomenological analysis
Hendricks‐Ferguson, 2015, USA [51]	Qualitative empirical phenomenology study	Pediatric oncology settings	14 nurses	Focus group discussions	Phenomenological analysis
Jucker, 2024, Switzerland [52]	Descriptive qualitative study	NICU and PICU ECMO center	21 nurses	Focus groups	Reflexive thematic analysis
Lewis, 2017, USA [53]	Qualitative narrative study	NICU	36 nurses	Written narratives	Thematic narrative analysis
Ma, 2021, China [54]	Descriptive qualitative study	Pediatric oncology wards	22 nurses	Semistructured interviews	Thematic analysis
MacDermott, 2014, and Keenan, 2016, Ireland [55, 56]	Qualitative descriptive study	Healthcare organization for people with intellectual disability	8 nurses	Semistructured interviews	Pragmatic qualitative data analysis
McCloskey, 2010, UK [57]	Qualitative study	Hospice, community and hospital specialist setting	18 nurses	Focus group discussions	Thematic content analysis
Nugent, 2022, Ireland [13]	Qualitative study	Pediatric ward	5 nurses	In‐depth semistructured interviews	Interpretive phenomenological analysis
Özsavran, 2026, Turkey [58]	Descriptive qualitative study	Pediatric intensive care unit	13 nurses	Semistructured interviews	Content analysis
Pant, 2026, UK [59]	Hermeneutic phenomenological study	NICU	8 healthcare personnel, including 5 nurses	Online semistructured in‐depth interviews	Hermeneutic phenomenological analysis
Papadatou, 1999, Greece [60]	Grounded theory study	Pediatric oncology and PICU	39 nurses	In‐depth semistructured interviews	Grounded theory‐informed qualitative analysis
Ramadan, 2025, Egypt [14]	Qualitative phenomenological study	NICU	40 nurses	In‐depth semistructured interviews and reflective journaling	Interpretative phenomenological analysis
Reid, 2013a, Scotland, UK [61]	Flexible qualitative design using grounded theory	Community/Home setting	7 nurses	Single face‐to‐face semistructured interviews	Distillation analysis
Reid, 2013b, Scotland, UK [62]	Phenomenological study	Rural community/Home setting	10 nurses	Semistructured interviews	Latent inductive content analysis
Roach, 2023, Oman [22]	Interpretive phenomenological analysis study	Pediatric hematology‐oncology units	11 nurses	Semistructured face‐to‐face interviews	Interpretative phenomenological analysis
Santos, 2014, Brazil [63]	Qualitative study	Pediatric wards	20 nursing professionals	Semistructured individual and group interviews	Content analysis
Shattnawi, 2022, Jordan [64]	Exploratory descriptive qualitative study	NICU	12 nurses	Semistructured face‐to‐face interviews	Thematic analysis
Stayer, 2016, USA [65]	Hermeneutic phenomenological study	PICU	12 nurses	Face‐to‐face interviews and field notes	Hermeneutic phenomenological analysis
Suryani, 2018, Indonesia [66]	Descriptive qualitative phenomenological study	Home care facility	10 nurses	Semistructured in‐depth interviews	Phenomenological analysis

*Note:* Participant totals include nursing participants only; companion reports were counted as one study.

Abbreviations: NICU = neonatal intensive care unit, PICU = pediatric intensive care unit, PPC = pediatric palliative care.

JBI appraisal indicated congruity with the research question, data collection, interpretation, and conclusions in all 37 studies (Q2, Q3, Q5, and Q10). Methodology–analysis congruity was supported in 33 studies, participants’ voices were adequately represented in all 37, and ethical conduct was supported in 35. Researcher positioning and influence were reported adequately in only 4 and 10 studies, respectively; philosophical–methodological congruity was unclear in 19. Item‐level ratings are shown in Figure 2.

**FIGURE 2:**
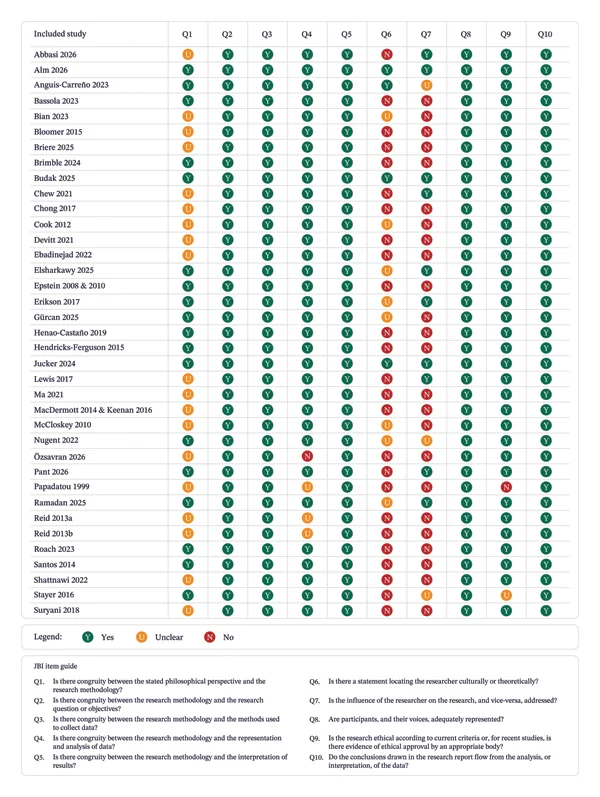
Quality appraisal results of included studies.

### 3.3. Main Findings of the Meta‐Synthesis

Four analytical themes and 18 descriptive subthemes were developed (Figure 3). Together, they describe how nurses sought to sustain compassionate care through meaningful work, negotiated relationships, responses to grief, and support. Compassionate professional presence offers an integrative interpretation of these efforts and the conditions shaping nurses’ emotional and therapeutic availability. Supporting Table 3 presents the GRADE‐CERQual evidence profile.

**FIGURE 3:**
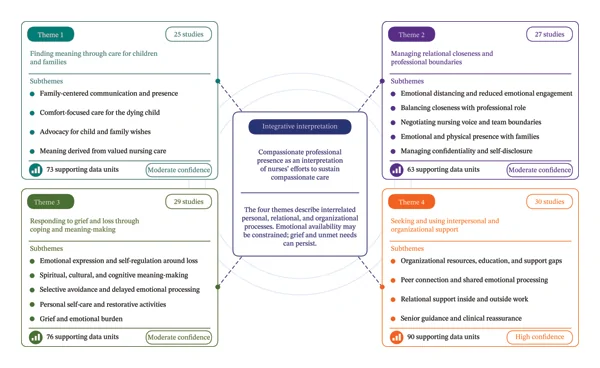
Themes, subthemes, and CERQual confidence.

### 3.4. Theme 1: Finding Meaning Through Care for Children and Families

This theme drew on 25 studies and 73 supporting data units (moderate confidence). Nurses described communication, comfort, presence, and advocacy as central to the care they sought to provide. Accounts of professional value and fulfillment emphasized the significance of their contribution to children and families, alongside frustration and distress when desired care was constrained.

#### 3.4.1. Subtheme 1.1: Family‐Centered Communication and Presence

Frequent bedside contact helped nurses develop trusting relationships and recognize families’ informational and emotional needs [51]. Nurses initiated conversations about clinical deterioration, adapted explanations to families’ understanding, and clarified treatment or comfort‐focused options to support parental decision‐making [35, 38, 46].

*“When you’re at cot-side for 12 hours… you build up this relationship very quickly with the parents… it’s the nurses that have got their relationship with them parents and who know that baby.”* [59].

*“I’d spent a lot of time talking with the parents after their last family meeting, explaining to them that it wasn’t just pulling the tube out… They could limit the care; they could limit the medications… They could specifically say that they wanted her comfortable*.*”* [46]

Nurses acknowledged the child’s individuality by using the child’s name and facilitating meaningful memories [37]. Accompaniment sometimes continued into bereavement through contact with parents and attendance at funerals [37, 44].

*“After the child’s death, although I was not in a good mood; at the request of the mother of the child, who was deeply saddened, I went to her house and stayed with her for hours until she calmed down.”* [44].

*“It was almost like we continued care for the family rather than for the patient.”* [37].

#### 3.4.2. Subtheme 1.2: Comfort‐Focused Care for the Dying Child

As children approached death, nurses emphasized relief of suffering and comfort through pharmacological and nonpharmacological measures [38, 66]. They described this care as a continuing responsibility when death was expected [35, 49, 50].

*“Making sure that the baby is as comfortable as possible and preventing any sort of like traumatic breathing or anything is helpful, because parents want to see that they are in fact allowing their baby to pass peacefully.”* [38].

*“I know the child will pass away despite receiving treatment and care, I see it as my final duty to the child.”* [49].

Nurses also sought to personalize the child’s final moments and honor feasible wishes alongside symptom relief [44, 65, 66].

*“It is important to me to make that last time for the family as personal as possible.”* [65].

#### 3.4.3. Subtheme 1.3: Advocacy for Child and Family Wishes

Nurses described advocacy as protecting children’s welfare and helping families express their priorities. Their accounts encompassed speaking on families’ behalf, supporting meaningful time together, and reflecting on whether invasive interventions could have been limited [53, 65].

*“This meeting is your opportunity to say what you need to say, and if you can’t say it, if you give me permission to say it for you, I’m more than happy to do that.”* [38].

*“In retrospect, I would have advocated for the infant to have less invasive intervention.”* [53].

*“I feel like I am in the middle of these situations as I see both perspectives, but I advocate for the welfare of the child.”* [65].

Advocacy also involved negotiating visiting restrictions and practical arrangements to accommodate children’s and families’ wishes [44, 59].

*“We reduced meeting restrictions… We eased some of the visiting restrictions for grandparents and other relatives.”* [44].

#### 3.4.4. Subtheme 1.4: Meaning Derived From Valued Nursing Care

Some nurses described their work as meaningful, emphasizing its professional value and the privilege of supporting families through loss [40, 45].

*“The job I’m doing right now might be the most important in the world.”* [40].

*“I hold the parents’ hands and show them how to cradle their baby for the last time. It’s painful but also a privilege to support them.”* [45].

### 3.5. Theme 2: Managing Relational Closeness and Professional Boundaries

This theme drew on 27 studies and 63 supporting data units (moderate confidence). Nurses negotiated emotional closeness, self‐protection, and professional responsibilities in relationships with families and colleagues. Accounts encompassed deliberate adjustments in involvement, efforts to remain present, and emotional numbness following repeated loss.

#### 3.5.1. Subtheme 2.1: Emotional Distancing and Reduced Emotional Engagement

Nurses regulated emotional closeness in response to repeated exposure to serious illness and death. Limiting personal involvement, setting aside emotions, and focusing on immediate tasks were ways of protecting themselves while continuing to provide care [19, 40].

*“I chose to distance myself from children’s personal lives, as knowing more makes their loss harder. This approach has helped me maintain my morale at work over the years.”* [19].

Some nurses described flexible emotional boundaries, using a protective barrier while allowing themselves to share emotion with families [34, 35].

*“The barrier, the armour has to be put on, it’s fine. But many times, if the situation is really touching, in my opinion it must be lived.”* [35].

Other nurses reported routine care accompanied by emotional numbness and difficulty sustaining compassion after repeated losses [14, 36].

*“I felt that care for dying children is part of the job, without any feeling, just routine care and rescue.”* [36].

#### 3.5.2. Subtheme 2.2: Balancing Closeness With Professional Role

Trust and attachment developed as nurses became familiar with children and families [34, 57]. Nurses calibrated their involvement to each family and situation while distinguishing professional warmth from personal friendship [39, 48].

*“You have to feel each situation out and see what’s appropriate for that family.”* [48].

*“… we do see it as part of our job, whereas you wouldn’t be ringing up that mum a couple of weeks later asking ‘are you free for coffee’ …”* [39].

Deepening relationships also heightened vulnerability to loss and concern about crossing professional boundaries [14, 48].

*“You always are afraid when you say you get close to a patient that you’re going beyond the appropriate boundaries.”* [48].

#### 3.5.3. Subtheme 2.3: Negotiating Nursing Voice and Team Boundaries

Nurses’ close and continuous contact with children and families provided detailed knowledge of their needs and preferences, yet this knowledge was sometimes overlooked in multidisciplinary decision‐making [37]. Nurses described frustration at being excluded from discussions and challenged decisions when they believed family perspectives required greater consideration [59].

*“We know the families better than anyone else, we develop a rapport, we share the experience with them, but that can be ignored.”* [37].

Communication with families also required nurses to negotiate the scope of nursing and medical responsibilities [36, 38]. Conflicting messages across teams left nurses uncertain about what they could explain and how to maintain a coherent account of the child’s care [43, 59].

*“When there is more than one team involved, and one is saying one thing, and the other is saying another, and you’re stuck in the middle…”* [43].

#### 3.5.4. Subtheme 2.4: Emotional and Physical Presence With Families

Nurses described presence as remaining physically and emotionally available during difficult moments. Quiet accompaniment and shared tears offered ways of staying connected with families when suffering and uncertainty were overwhelming [12, 34, 35].

*“You have the courage to stay, even when everything is ugly and chaotic and overwhelming.”* [12].

*“I happened to cry with parents. But this sharing is beautiful, they feel you close, they know you are there.”* [35].

Experience informed nurses’ judgments about when to speak, remain silent, or follow the family’s pace [12].

*“There’s no manual for this. You just know, from having been there before.”* [12].

#### 3.5.5. Subtheme 2.5: Managing Confidentiality and Self‐Disclosure

Nurses used selective self‐disclosure to build rapport while keeping relationships centered on children and families [39]. Relationships extending into social media and close‐knit communities required particular care over confidentiality, personal disclosure, and the boundaries of nurses’ private lives [48, 49].

*“You do share some things don’t you… as a way to connect.”* [39].

*“I hesitate to share happy moments from my social life, such as my son’s birthday, on social media because I don’t want mothers whose children are in the end-of-life stage to see it and feel upset.”* [49].

### 3.6. Theme 3: Responding to Grief and Loss Through Coping and Meaning‐Making

This theme drew on 29 studies and 76 supporting data units (moderate confidence). Grief occurred before, during, and after death. Nurses responded through emotional expression and regulation, spiritual and cognitive meaning‐making, selective avoidance, and self‐care. The timing and perceived relief of these responses varied, with some nurses describing persistent emotional burden.

#### 3.6.1. Subtheme 3.1: Emotional Expression and Self‐Regulation Around Loss

Nurses described moving between expressing sadness and containing distress to meet immediate care responsibilities. Crying offered some nurses a sense of relief [19, 54].

*“After crying, I felt better.”* [54].

Nurses also sought quiet spaces or briefly stepped back to manage distress after difficult events [36, 65].

*“It was all self-regulating, finding a quiet corner, not wanting to ask for help from others, who did not necessarily understand because they have not experienced it.”* [36].

Redirecting attention toward the remaining family’s needs provided another way of managing grief while continuing care [36, 42].

*“I think my coping strategy is this little mind shift that I now have to take care of the living … the (remaining) family …”* [42].

#### 3.6.2. Subtheme 3.2: Spiritual, Cultural, and Cognitive Meaning‐Making

Nurses drew on faith, prayer, and beliefs about destiny or an afterlife to interpret death and accept outcomes beyond their control [19, 41]. Spiritual reassurance coexisted with continuing pain in some accounts [41].

*“Death is a reality that everyone faces, and each person’s destiny is in God’s hands. This belief gives me peace of mind.”* [19].

Nurses also interpreted death as release from unremitting suffering and reflected on the fragility and value of life [19, 37, 40].

*“I can start to see death more as a form of deliverance. It seems like a way of deliverance for both the child and the family.”* [40].

#### 3.6.3. Subtheme 3.3: Selective Avoidance and Delayed Emotional Processing

Nurses avoided photographs, memories, or conversations that reactivated distress [19, 54]. They postponed emotional expression until after work to remain composed while caring for other children and families [34, 49].

*“I prefer not to revisit memories… I try to avoid taking photos with them to prevent their memories from being evoked.”* [19].

*“… if I have to cry, I cry … I prefer to cry at home… but when I am at work, I have to be there… and hold on like a champion…”* [34].

Distress sometimes resurfaced after work, and some nurses recognized their need for support only later [13, 56].

*“Looking back however, I did need some support.”* [56].

#### 3.6.4. Subtheme 3.4: Personal Self‐Care and Restorative Activities

Nurses used exercise and everyday activities to release tension and regain a sense of balance [19, 34, 38]. Reflective journaling offered a way to process difficult experiences [14].

*“To improve my mood, I go swimming or for a walk on weekends.”* [19].

*“I do physical exercise… it’s a way of releasing tension and adrenaline… I clear my head by running or cycling…”* [34].

*“I keep a journal to process my experiences.”* [14].

#### 3.6.5. Subtheme 3.5: Grief and Emotional Burden

Sadness, guilt, self‐questioning, and enduring attachment arose before, during, or after a child’s death [13, 36, 43]. Some nurses carried vivid memories and unresolved distress into their personal lives, and repeated loss left others feeling emotionally exhausted [48, 57].

*“When you have been through that, you feel like you’ve been actually run over by a truck… you come out and you’re not normal, you can’t be normal… You go home that night and you are thinking about them.”* [43].

*“I do feel quite burnt out you know. How much compassion have I left?”* [57].

### 3.7. Theme 4: Seeking and Using Interpersonal and Organizational Support

This theme drew on 30 studies and 90 supporting data units (high confidence). Nurses sought understanding, clinical guidance, and practical resources through peer, senior, personal, and organizational support. Their accounts described variation in access, uptake, and perceived usefulness, including unmet needs and experiences of inaccessible or unhelpful provision.

#### 3.7.1. Subtheme 4.1: Organizational Resources, Education, and Support Gaps

Staffing shortages and heavy workloads limited nurses’ time for emotional connection with children and families [14, 19]. Nurses also reported insufficient preparation for end‐of‐life care and called for continuing education in communication and grief management, together with institutional psychological support for distress extending beyond work [34, 64].

*“You want to be there emotionally, but you don’t have the time.”* [14].

*“I wish that the hospital provided us with courses on communication skills, delivered by a specialist in psychological health who can give us guidelines and coping techniques to ease our grieving process after the death of a child.”* [64].

*“I would appreciate the presence of a psychologist at the center to help me manage the knot in my stomach with which I have gone home, some days because I don’t know how to deal with the bad (…).”* [34].

Emotional strain and unmet support needs also featured in accounts of seeking a transfer or leaving a clinical role [36, 43].

*“I think my move from [the ward] was a massive part of that, because it was starting to affect my personal life, so I had to move on. And that’s not good because I loved the work, but I think it pushes people away from the work because the support isn’t there.”* [43].

#### 3.7.2. Subtheme 4.2: Peer Connection and Shared Emotional Processing

Colleagues were valued for understanding the emotional impact of caring for dying children through shared clinical experience [19, 34]. Informal conversations, listening, reassurance, and debriefing offered opportunities to express distress and feel less isolated [13, 35].

*“It is about your colleagues supporting each other because they are the only ones that know what you’ve been through.”* [13].

Peer exchanges combined emotional support with discussion of clinical concerns and difficult care decisions [38, 59].

*“Our biggest supports are colleagues… we rely on each other, we run things by each other, we talk to each other about it whether it’s a medical thing or a social thing.”* [38].

#### 3.7.3. Subtheme 4.3: Relational Support Inside and Outside Work

Nurses sought comfort from partners, family members, friends, and wider professional networks [34, 54, 65]. These relationships provided opportunities to discuss experiences that continued to affect them after work.

*“I try not to take it home, but I do! There are many times I talk to my husband about a horrible case.”* [65].

Support outside work could be limited by others’ difficulty understanding or tolerating accounts of a child’s death [19].

*“My spouse is sensitive to illness and pain, and gets upset at the sight of blood or hearing news about death. They ask me not to talk about these issues.”* [19].

#### 3.7.4. Subtheme 4.4: Senior Guidance and Clinical Reassurance

Experienced nurses offered practical assistance, modeled communication and care, and reassured less experienced colleagues [51, 64]. Following a child’s death, some nurses received affirming responses from leaders, while others sought reassurance about their responsibility for the outcome [36].

*“When we have a baby in the EOL stage, the senior nurse stays with us and help us.”* [64].

*“The head nurse came and hugged me, and she whispered to me, ‘You were doing well’.”* [36].

## 4. Discussion

This synthesis indicates that nurses’ efforts to sustain compassionate care are shaped by the interaction between valued care, relational involvement, responses to loss, and the conditions of practice. The four themes offer complementary perspectives: Meaning in care explains what nurses value; boundary management concerns how they negotiate involvement; grief and coping describe responses to the emotional demands of continuing care; and support identifies resources and constraints surrounding these efforts. Compassionate professional presence integrates these findings as nurses’ efforts to remain therapeutically and emotionally available. Building on reviews of palliative‐care experiences, grief, and coping [25–27], the synthesis highlights how organizational decisions shape both the care nurses can provide and the emotional demands they must manage.

Meaning was grounded in the value nurses attributed to their contribution, particularly comfort, communication, and support for families when cure was no longer possible [38, 65]. This finding is consistent with accounts of relational nursing care, a good death for the child, and the rewards of palliative work [3, 67]. The themes together suggest that deep involvement in valued care could heighten vulnerability to loss, while constraints on that care could generate frustration. Meaning and distress can therefore coexist within the same caregiving relationship. This connects staff well‐being to nurses’ ability to act on their clinical and relational responsibilities.

Boundary management emerged as ongoing judgment about appropriate involvement. Shared emotion, selective self‐disclosure, and adjustments in closeness were ways nurses attended to families while preserving professional responsibilities [19, 39, 48]. These findings complement broader literature on trust, professional intimacy, and self‐disclosure in nursing relationships [68, 69]. The synthesis also distinguishes deliberate distancing from emotional numbness after repeated loss. This distinction matters for supervision: Similar outward behavior may reflect an intentional boundary or difficulty remaining emotionally available. Reflective discussion offers an opportunity to clarify the nurse’s needs within the particular relationship and clinical situation. The Emotional Labor Model in Pediatric Nursing offers a lens for interpreting this negotiation: Nurses manage their own emotional responses while attending to those of the child and family [16]. Active listening, quiet accompaniment, and shared emotion thus involve purposeful emotional work that supports therapeutic presence. Within palliative care, this relational work accompanies interventions addressing physical pain and emotional and spiritual distress [3, 18]. The model connects family‐centered care with the emotional demands of maintaining proximity and availability. This perspective supports education in emotional regulation and reflective supervision as approaches to addressing these demands and burnout risk [18].

Nursing voice reveals a further connection between emotional labor and organizational ethics. Nurses’ detailed knowledge of families could be overlooked, while conflicting team messages left them explaining care without a coherent shared plan [37, 38, 59]. The resulting frustration suggests that distress arises partly from how responsibilities and influence are distributed within teams. Bedside nurses’ participation in goals‐of‐care and ethics discussions is therefore relevant to both family‐centered decisions and staff well‐being. Leadership that invites nursing perspectives and clarifies shared decisions may ease the burden of managing unresolved team disagreements in encounters with families.

Coping with loss was shaped by timing and changing demands. Nurses moved between emotional expression, temporary avoidance, and attention to immediate care responsibilities, consistent with Papadatou’s account of oscillation in professional grief [70]. Meaning‐making after loss involved interpreting death and its place in life, whereas meaning in care concerned the value of nurses’ contribution. Accounts of exercise and reflection align with literature describing self‐care as an ongoing practice and its associations with professional well‐being [71, 72]. Within this synthesis, composure during a shift could coexist with distress afterward and later recognition of support needs [34, 54]. Support offered beyond the immediate postdeath period may therefore better match nurses’ changing needs.

Support was valued when it combined understanding of the emotional experience with practical help and clinical reassurance. Peer exchanges and senior guidance connected emotional processing with discussion of care decisions [38, 59]. This helps explain why access, perceived relevance, and trusted relationships mattered alongside the availability of formal services. Accounts of leaving a valued role amid persistent distress and inadequate support make these needs relevant to workforce sustainability [36, 43]. Together with broader evidence linking work environments and leadership to nurses’ well‐being [73, 74], the findings suggest that supportive cultures are expressed through everyday responses to distress and practical access to assistance. Storytelling, debriefing, education, and mentoring described in other studies offer options for local development and evaluation [6, 73].

### 4.1. Limitations

The synthesis relied on primary authors’ selection, interpretation, and reporting of participants’ accounts. Limited quotations and insufficient contextual or reflexive detail may have constrained analytic depth. Variation in clinical settings, nursing roles, palliative‐care provision, and organizational contexts limited comparisons across neonatal and pediatric intensive care, oncology, hospice, and community settings. Uneven representation of countries and healthcare systems may also limit transferability, particularly to resource‐constrained settings where staffing, specialist services, and cultural expectations differ. The four themes were closely interconnected, with some accounts informing more than one theme. The framework therefore represents overlapping processes whose temporal and causal relationships require further investigation. Finally, the findings reflect nurses’ reported experiences and perceived support needs; the effects of specific management approaches on staff well‐being, care delivery, and retention require evaluation.

## 5. Conclusions

This review connects nurses’ experiences of sustaining compassionate care with the personal, relational, and organizational processes surrounding that work. Nurses found value in caring for children and families, negotiated emotional and professional boundaries, responded to grief over time, and sought support that was accessible and meaningful to them. These efforts coexisted with vulnerability, unresolved grief, and constrained emotional availability. Compassionate professional presence captures this ongoing work of remaining available within changing clinical and relational demands. The synthesis highlights nursing management’s responsibility to create conditions in which valued care, staff support, and participation in decisions can be sustained together.

## 6. Implications for Nursing Management

Managers should protect time and resources for the communication, comfort, advocacy, and family contact that nurses valued. Reports of exclusion from decisions support routine bedside nursing input into goals‐of‐care and ethics discussions, with clear team roles and consistent communication with families. Boundary tensions and emotional demands warrant case‐based reflective supervision with experienced facilitators, offering confidential individual discussion or voluntary peer reflection according to staff preferences. Leaders should acknowledge grief, invite concerns, and facilitate access to senior clinical advice and confidential psychological support. Because support needs sometimes became apparent later, follow‐up should extend beyond the immediate aftermath of a child’s death. Staffing coverage and protected participation time are needed to make these opportunities accessible. Feedback on access, uptake, perceived usefulness, and unmet needs should guide adjustments to support.

**Nomenclature**
CERQual: Confidence in the Evidence from Reviews of Qualitative Research
ENTREQ: Enhancing Transparency in Reporting the Synthesis of Qualitative Research
JBI: Joanna Briggs Institute
NICU: Neonatal intensive care unit
PICU: Pediatric intensive care unit
PRISMA: Preferred Reporting Items for Systematic Reviews and Meta‐Analyses
PROSPERO: International Prospective Register of Systematic Reviews

## Author Contributions

Yuan Li: conceptualization, methodology, validation, data extraction, data synthesis, quality appraisal, writing–original draft, and writing–review and editing. Shiqi Luo: methodology, study design, literature search, study selection, quality appraisal, writing–original draft, and writing–review and editing. Xia Li: literature screening, data extraction, and data curation. Hanmei Peng: validation, methodology, and data analysis. Yanling Hu: supervision, project administration, and writing–review and editing. Qiong Chen: supervision, project administration, and writing–review and editing. All authors critically revised the manuscript.

## Funding

This work was supported by the Sichuan Medical Association (Grant Number: Q2024011), the Chengdu Science and Technology Bureau (Grant Number: 2024‐YF05‐00503‐SN), and the Natural Science Foundation of Sichuan Province (2026NSFSC1721).

## Disclosure

All authors approved the final version and agreed to be accountable for all aspects of the work. The funders had no role in study design, data collection and analysis, decision to publish, or preparation of the manuscript.

## Ethics Statement

This study did not involve the recruitment of human participants or the collection of primary data and therefore did not require ethical approval or informed consent.

## Consent

The authors have nothing to report.

## Conflicts of Interest

The authors declare no conflicts of interest.

## Supporting Information

Additional supporting information can be found online in the Supporting Information section.

## Supporting information


**Supporting Information** The supporting file provides additional materials supporting this review, including database‐specific search strategies and search results (Supporting Material A), full‐text exclusions and reasons for exclusion (Supporting Table 1), characteristics of the included studies (Supporting Table 2), and the GRADE‐CERQual evidence profile (Supporting Table 3).

## Data Availability

All data generated or analyzed during this review are included in this published article and its supporting information files.
