# Supplementary material for: Sustaining Compassionate Care in Pediatric and Neonatal Palliative Care: A Qualitative Meta‐Synthesis of Nurses’ Experiences Amid Uncertainty and Loss
Source: J Nurs Manag. 2026 Nov 1;2026:5143378. doi: 10.1155/jonm/5143378 (PMC13725121; doi:10.1155/jonm/5143378)
Supplement: Supplementary file 1 [file JONM-2026-5143378-s001.docx]

# **Supplementary Information**

[**Content**	1](#_Toc7373)

[**Supplementary Material A:** Search strategies and results (Updated: 24 September 2026)	2](#_Toc25875)

[**Supplementary Table 1.** Full-text exclusions and reasons	8](#_Toc12802)

[**Supplementary Table 2.** Characteristics of the included studies (n = 37)	18](#_Toc29086)

[**Supplementary Table 3.** GRADE-CERQual evidence profile	31](#_Toc14462)

**Supplementary Material A:** Search strategies and results (Updated: 24 September 2026)

PubMed (n = 627)

#1: "Nurses"[Mesh] OR "Nursing Staff"[Mesh] OR "Neonatal Nursing"[Mesh] OR "Pediatric Nursing"[Mesh] OR nurs*[Title/Abstract]

#2: "Pediatrics"[Mesh] OR "Child"[Mesh] OR "Infant, Newborn"[Mesh] OR "Intensive Care Units, Neonatal"[Mesh] OR "Intensive Care Units, Pediatric"[Mesh] OR pediatric*[Title/Abstract] OR paediatric*[Title/Abstract] OR child*[Title/Abstract] OR adolescent*[Title/Abstract] OR neonat*[Title/Abstract] OR newborn*[Title/Abstract] OR infant*[Title/Abstract] OR NICU[Title/Abstract] OR PICU[Title/Abstract]

#3: "Palliative Care"[Mesh] OR "Terminal Care"[Mesh] OR "Hospice Care"[Mesh] OR palliative[Title/Abstract] OR "terminal care"[Title/Abstract] OR hospice*[Title/Abstract] OR "end of life"[Title/Abstract] OR end-of-life[Title/Abstract] OR "life-limiting"[Title/Abstract]

#4: "Qualitative Research"[Mesh] OR "qualitative"[Title/Abstract] OR interview*[Title/Abstract] OR "focus group"[Title/Abstract] OR "focus groups"[Title/Abstract] OR "content analysis"[Title/Abstract] OR "thematic analysis"[Title/Abstract] OR phenomenolog*[Title/Abstract] OR ethnograph*[Title/Abstract] OR "grounded theory"[Title/Abstract] OR "mixed method"[Title/Abstract] OR "mixed methods"[Title/Abstract]

#5: #1 AND #2 AND #3 AND #4

Web of Science (n = 620)

#1: TS = (nurs* OR "nursing staff" OR "neonatal nursing" OR "pediatric nursing" OR "paediatric nursing")

#2: TS = (pediatric* OR paediatric* OR child* OR adolescent* OR neonat* OR newborn* OR infant* OR NICU OR PICU OR "neonatal intensive care unit*" OR "pediatric intensive care unit*" OR "paediatric intensive care unit*")

#3: TS = ("palliative care" OR "terminal care" OR "hospice care" OR palliative OR hospice* OR "end of life" OR "end-of-life" OR "life-limiting")

#4: TS = ("qualitative research" OR qualitative OR interview* OR "focus group*" OR "content analysis" OR "thematic analysis" OR phenomenolog* OR ethnograph* OR "grounded theory" OR "mixed method*")

#5: #1 AND #2 AND #3 AND #4

CINAHL (n = 548)

S1: (MH "Nurses+" OR MH "Nursing Staff+" OR MH "Neonatal Nursing" OR MH "Pediatric Nursing" OR TI (nurs*) OR AB (nurs*))

S2: (MH "Child+" OR MH "Infant+" OR MH "Pediatrics+" OR MH "Intensive Care Units, Neonatal" OR MH "Intensive Care Units, Pediatric" OR TI (pediatric* OR paediatric* OR child* OR adolescent* OR neonat* OR newborn* OR infant* OR NICU OR PICU) OR AB (pediatric* OR paediatric* OR child* OR adolescent* OR neonat* OR newborn* OR infant* OR NICU OR PICU))

S3: (MH "Palliative Care" OR MH "Terminal Care" OR MH "Hospice Care" OR TI ("palliative care" OR "terminal care" OR "hospice care" OR palliative OR hospice* OR "end of life" OR end-of-life OR "life-limiting") OR AB ("palliative care" OR "terminal care" OR "hospice care" OR palliative OR hospice* OR "end of life" OR end-of-life OR "life-limiting"))

S4: (MH "Qualitative Studies+" OR MH "Interviews+" OR MH "Focus Groups" OR MH "Grounded Theory" OR MH "Phenomenological Research" OR TI (qualitative OR interview* OR "focus group*" OR "content analysis" OR "thematic analysis" OR phenomenolog* OR ethnograph* OR "grounded theory" OR "mixed method*") OR AB (qualitative OR interview* OR "focus group*" OR "content analysis" OR "thematic analysis" OR phenomenolog* OR ethnograph* OR "grounded theory" OR "mixed method*"))

S5: S1 AND S2 AND S3 AND S4

Embase (n = 683)

#1: 'nurse'/exp OR 'nursing staff'/exp OR 'pediatric nursing'/exp OR nurs*:ti,ab,kw OR 'neonatal nursing':ti,ab,kw OR 'pediatric nursing':ti,ab,kw

#2: 'pediatrics'/exp OR 'child'/exp OR 'newborn'/exp OR 'neonatal intensive care unit'/exp OR 'pediatric intensive care unit'/exp OR pediatric*:ti,ab,kw OR paediatric*:ti,ab,kw OR child*:ti,ab,kw OR adolescent*:ti,ab,kw OR neonat*:ti,ab,kw OR newborn*:ti,ab,kw OR infant*:ti,ab,kw OR nicu:ti,ab,kw OR picu:ti,ab,kw

#3: 'palliative therapy'/mj OR 'terminal care'/mj OR 'hospice care'/mj OR 'palliative care':ti OR 'terminal care':ti OR 'hospice care':ti OR palliative:ti OR hospice*:ti OR 'end of life':ti OR 'end-of-life':ti OR 'life-limiting':ti

#4: 'qualitative research'/exp OR 'interview'/exp OR 'focus group'/exp OR 'qualitative analysis'/exp OR qualitative:ti,ab,kw OR interview*:ti,ab,kw OR 'focus group*':ti,ab,kw OR 'content analysis':ti,ab,kw OR 'thematic analysis':ti,ab,kw OR phenomenolog*:ti,ab,kw OR ethnograph*:ti,ab,kw OR 'grounded theory':ti,ab,kw OR 'mixed method*':ti,ab,kw

#5: #1 AND #2 AND #3 AND #4

Scopus (n = 1075)

( TITLE-ABS-KEY ( nurs* OR "nursing staff" OR "neonatal nursing" OR "pediatric nursing" OR "paediatric nursing" OR "clinical nurse*" OR "registered nurse*" ) ) AND ( TITLE-ABS-KEY ( pediatric* OR paediatric* OR child* OR adolescent* OR neonat* OR newborn* OR infant* OR nicu OR picu OR "neonatal intensive care unit*" OR "pediatric intensive care unit*" OR "paediatric intensive care unit*" ) ) AND ( TITLE-ABS-KEY ( "palliative care" OR "terminal care" OR "hospice care" OR palliative OR hospice* OR "end of life" OR "end-of-life" OR "life-limiting" ) ) AND ( TITLE-ABS-KEY ( "qualitative research" OR qualitative OR interview* OR "focus group*" OR "content analysis" OR "thematic analysis" OR phenomenolog* OR ethnograph* OR "grounded theory" OR "mixed method*" ) )

PsycINFO (n = 294)

S1:DE "Nurses" OR DE "Pediatric Nurses" OR TI (nurs* OR "nursing staff" OR "neonatal nursing" OR "pediatric nursing") OR AB (nurs* OR "nursing staff" OR "neonatal nursing" OR "pediatric nursing")

S2:DE "Pediatrics" OR DE "Neonatology" OR DE "Infants" OR DE "Children" OR TI (pediatric* OR paediatric* OR child* OR adolescent* OR neonat* OR newborn* OR infant* OR nicu OR picu) OR AB (pediatric* OR paediatric* OR child* OR adolescent* OR neonat* OR newborn* OR infant* OR nicu OR picu)

S3:DE "Palliative Care" OR DE "Terminally Ill Patients" OR DE "Hospice" OR DE "Death and Dying" OR DE "Grief" OR DE "Bereavement" OR TI ("palliative care" OR "terminal care" OR "hospice care" OR palliative OR hospice* OR "end of life" OR "end-of-life" OR "life-limiting") OR AB ("palliative care" OR "terminal care" OR "hospice care" OR palliative OR hospice* OR "end of life" OR "end-of-life" OR "life-limiting")

S4:DE "Qualitative Research" OR DE "Interviews" OR DE "Phenomenology" OR DE "Grounded Theory" OR TI (qualitative OR interview* OR "focus group" OR "focus groups" OR "content analysis" OR "thematic analysis" OR phenomenolog* OR ethnograph* OR "grounded theory" OR "mixed method" OR "mixed methods") OR AB (qualitative OR interview* OR "focus group" OR "focus groups" OR "content analysis" OR "thematic analysis" OR phenomenolog* OR ethnograph* OR "grounded theory" OR "mixed method" OR "mixed methods")

S6:S1 AND S2 AND S3 AND S4

# **Supplementary Table 1.** Full-text exclusions and reasons

| **Reason for exclusion** | **Database search (n)** | **Reference-list screening (n)** | **Total (n)** | **Example references** |
| --- | --- | --- | --- | --- |
| Ineligible phenomenon of interest | 60 | 17 | 77 | [1-77] |
| Ineligible context | 11 | 8 | 19 | [78-96] |
| Ineligible study design | 4 | 6 | 10 | [97-106] |
| Insufficient extractable qualitative findings | 22 | 7 | 29 | [107-135] |
| Ineligible publication type | 5 | 0 | 5 | [136-140] |

*Note:* Exclusion categories correspond to phenomenon of interest, context, study design, availability of relevant qualitative findings, and publication type. “Insufficient extractable qualitative findings” refers to reports without separable nursing data relevant to the review question.

**References**

1.	Al Mutair A, Al Ammary M, Brooks LA, Bloomer MJ. Supporting Muslim families before and after a death in neonatal and paediatric intensive care units. *Nursing in critical care*. 2019;24(4):192-200. doi:10.1111/nicc.12434

2.	Arciprete EGB, Michelson KN, Feder SL, Marr JW, Al-Roub N, Gouda SR, et al. Noticing and responding to end-of-life suffering in the PICU: A co-designed conceptual framework for nursing practice. *Intensive and Critical Care Nursing*. 2026;96. doi:10.1016/j.iccn.2026.104498

3.	Banerjee SC, Manna R, Coyle N, Shen MJ, Pehrson C, Zaider T, et al. Oncology nurses' communication challenges with patients and families: A qualitative study. *Nurse education in practice*. 2016;16(1):193-201. doi:10.1016/j.nepr.2015.07.007

4.	Barrington L. Aqualitative exploration of nurses perceptions on palliative care for children with a life-limiting cardiac diagnosis, in a tertiary cardiac centre. *Cardiology in the Young*. 2025;35:S336-S7. doi:10.1017/S1047951125101674

5.	Beckstrand RL, Isaacson RF, Macintosh JLB, Luthy KE, Eden L. NICU Nurses' Suggestions for Improving End-of-Life Care Obstacles. *Journal of Neonatal Nursing*. 2019;25(1):32-6. doi:10.1016/j.jnn.2018.08.004

6.	Beringer AJ, Eaton NM, Jones GL. Providing a children′s palliative care service in the community through fixed-term grants:: the staff perspective. *Child Care Health and Development*. 2007;33(5):619-24. doi:10.1111/j.1365-2214.2007.00762.x

7.	Bloomer MJ, Endacott R, Copnell B, O'Connor M. 'Something normal in a very, very abnormal environment'--Nursing work to honour the life of dying infants and children in neonatal and paediatric intensive care in Australia. *Intensive & critical care nursing*. 2016;33:5-11. doi:10.1016/j.iccn.2015.09.001

8.	Borhani F, Abbaszadeh A, Mohsenpour M, Asadi N. Lived experiences of pediatric oncology nurses in Iran. *Iranian journal of nursing and midwifery research*. 2013;18(5):349-54.

9.	Boyd D, Merkh K, Rutledge DN, Randall V. Nurses' perceptions and experiences with end-of-life communication and care. *Oncology nursing forum*. 2011;38(3):E229-39. doi:10.1188/11.Onf.E229-e239

10.	Boztepe H, Kerimoğlu Yıldız G. Nurses perceptions of barriers to implementing family-centered care in a pediatric setting: A qualitative study. *Journal for specialists in pediatric nursing : JSPN*. 2017;22(2). doi:10.1111/jspn.12175

11.	Camilo BHN, Serafim TC, Salim NR, Andreato Á MO, Roveri JR, Misko MD. Communication of bad news in the context of neonatal palliative care: experience of intensivist nurses. *Rev Gaucha Enferm*. 2022;43:e20210040. doi:10.1590/1983-1447.2022.20210040

12.	Cherer EdQ, Quintana AM, Pinheiro UMS. Sofrimento e Libertação: Significações Sobre a Morte na UTI Pediátrica. *PSICO*. 2013;44(4):482-9.

13.	Citak EA, Toruner EK, Gunes NB. Exploring communication difficulties in pediatric hematology: oncology nurses. *Asian Pac J Cancer Prev*. 2013;14(9):5477-82. doi:10.7314/apjcp.2013.14.9.5477

14.	Connor EO, Corcoran Y. Caring for a child with a life limiting condition: The experiences of nurses in an intellectual disability service provider. *J Intellect Disabil*. 2022;26(4):938-53. doi:10.1177/17446295211018588

15.	Costa Dias TK, da Silva Reichert AP, Evangelista CB, de Souza Batista PS, da Silva Buck EC, de Sá França JRF. Nurses assistance to children in palliative care: a study in the light of Jean Watson's theory. *Anna Nery School Journal of Nursing / Escola Anna Nery Revista de Enfermagem*. 2023;27:1-7. doi:10.1590/2177-9465-EAN-2021-0512en

16.	Dalcali BK, Can S, Durgun H. Emotional Responses of Neonatal Intensive Care Nurses to Neonatal Death. *Omega-Journal of Death and Dying*. 2022;85(2):497-513. doi:10.1177/0030222820971880

17.	Daniels GM, Tefera R, Martinez I, Buckingham SI, Metrock LK, Ramini S, et al. Early Palliative Care Is Feasible and Acceptable in Pediatric Neuro-Oncology. *Pediatric blood & cancer*. 2026:e70669. doi:10.1002/1545-5017.70669

18.	Daudt Fernandes V, de Sá Neto JA, Andrade Coutinho KA, Teixeira Reis A, Santos Santana da Silva AC. Nursing team's conceptions about palliative care in newborns. *UERJ Nursing Journal / Revista Enfermagem UERJ*. 2021;29:1-6. doi:10.12957/reuerj.2021.57257

19.	Davies B, Clarke D, Connaughty S, Cook K, MacKenzie B, McCormick J, et al. Caring for dying children: nurses' experiences. *Pediatric nursing*. 1996;22(6):500-7.

20.	de Sá França JRF, da Costa SFG, da Nóbrega MML, Lopes MEL. Palliative care to child with cancer. *Revista Enfermagem*. 2013;21(SPEC.ISSUE 2):779-84.

21.	de Souza LF, Misko MD, Silva L, Poles K, dos Santos MR, Bousso RS. Dignified death for children: perceptions of nurses from an oncology unit. *Revista Da Escola De Enfermagem Da Usp*. 2013;47(1):30-7. doi:10.1590/s0080-62342013000100004

22.	Di Kachlová MS, Bužgová R. Barriers to the provision of palliative care in neonatology from the perspective of nurses. *Pediatrie pro Praxi*. 2023;24(4):269-71. doi:10.36290/ped.2023.070

23.	Docherty SL, Miles MS, Brandon D. Searching for "the dying point:" providers' experiences with palliative care in pediatric acute care. *Pediatric nursing*. 2007;33(4):335-41.

24.	Duarte MDC, Glanzner CH, Bagatini MMC, da Silva DG, de Mattos LG. Pleasure and suffering in the work of nurses at the oncopediatric hospital unit: qualitative research. *Revista brasileira de enfermagem*. 2021;74. doi:10.1590/0034-7167-2020-0735

25.	Ebadinejad Z, Rassouli M, Fakhr Movahedi A. Exploration of the Strategies of Iranian Nurses in Providing Palliative Care to Children with Cancer: A Qualitative Study. *Florence Nightingale journal of nursing*. 2023;31(Supp1):S1-s6. doi:10.5152/fnjn.2023.0024

26.	El Ali M, O'Neill J, Gillam L. Paediatric nurses' personal accounts of being told not to disclose information to children with serious illness—An interpretative phenomenological study. *Journal of advanced nursing*. 2025;81(7):4067-84. doi:10.1111/jan.16596

27.	Engel M, van Zuylen L, van der Ark A, van der Heide A. Palliative care nurse champions' views on their role and impact: a qualitative interview study among hospital and home care nurses. *BMC palliative care*. 2021;20(1):34. doi:10.1186/s12904-021-00726-1

28.	Evered JA. Social Relationships in Nurses' Caring of Adolescents and Emerging Adults With Life-Limiting Illnesses or Injuries. *Qualitative health research*. 2021;31(3):578-89. doi:10.1177/1049732320978207

29.	Falck AJ, Moorthy S, Hussey-Gardner B. Perceptions of Palliative Care in the NICU. *Advances in neonatal care : official journal of the National Association of Neonatal Nurses*. 2016;16(3):191-200. doi:10.1097/anc.0000000000000301

30.	Ferrell B, Wittenberg E, Battista V, Walker G. Nurses' Experiences of Spiritual Communication with Seriously III Children. *Journal of palliative medicine*. 2016;19(11):1166-70. doi:10.1089/jpm.2016.0138

31.	Figueiredo de Sá França JR, Fatima Geraldo da Costa S, Lopes MEL, Miriam Lima da Nóbrega M, Sátiro Xavier de França I. The importance of communication in pediatric oncology palliative care: Focus on humanistic nursing theory. *Revista Latino-Americana de Enfermagem*. 2013;21(3):780-6. doi:10.1590/S0104-11692013000300018

32.	Fontaine E, Wendland J. Analyse de la relation soignant-bébé et du deuil des soignants en néonatologie. *Devenir*. 2015;27(1):31-52. doi:10.3917/dev.151.0031

33.	Funes MM, Moraes MW, Cunha M, Almeida FA. Caring for cancer patients facing death: nurse's perception and experience. *Revista brasileira de enfermagem*. 2020;73Suppl 5(Suppl 5):e20190686. doi:10.1590/0034-7167-2019-0686

34.	Furingsten L, Sjögren R, Forsner M. Ethical challenges when caring for dying children. *Nursing ethics*. 2015;22(2):176-87. doi:10.1177/0969733014533234

35.	Galanti T, Santoriello M, Cortini M, Di Tullio E, Di Febbo A, Fantinelli S, et al. Structural and Organizational Dimensions of Compassion Fatigue in Pediatric Oncology Nursing: A Qualitative Study. *International journal of environmental research and public health*. 2026;23(8). doi:10.3390/ijerph23080981

36.	Ghaljeh M, Mardani-Hamooleh M, Pezaro S. Nurses' perceptions of compassionate care in pediatric oncology: a qualitative interview study. *BMC Health Serv Res*. 2024;24(1):1165. doi:10.1186/s12913-024-11661-1

37.	González-Gil MT, Alcolea-Cosín MT, Pérez-García S, Luna-Castaño P, Torrent-Vela S, Piqueras-Rodríguez P, et al. Children's visits to the paediatric intensive care unit from the nurses' experience. *Enferm Intensiva (Engl Ed)*. 2021;32(3):133-44. doi:10.1016/j.enfie.2020.06.002

38.	Granek L, Hasan F, Johnston D, Lysecki D, van Zanten SV, Rapoport A, et al. The Influence of Emotions on Referral Practices to Pediatric Palliative Care Services in Oncology Settings in Canada. *Qualitative Psychology*. 2026. doi:10.1037/qup0000364

39.	Granek L, Hasan F, Lysecki D, Johnston D, Veldhuijzen van Zanten S, Datoo N, et al. Systems Theory Model to Understand the Barriers and Facilitators to Palliative Care Referral for Pediatric Oncology Patients in Canada. *JCO Oncology Practice*. 2026. doi:10.1200/OP-25-00761

40.	Granek L, van Zanten SV, Widger K, Wiener L, Johnston D, Hasan F, et al. Communication Barriers and Facilitators to Specialist Pediatric Palliative Care Involvement in Pediatric Oncology Settings Across Canada. *Psycho-oncology*. 2026;35(8). doi:10.1002/pon.70568

41.	Hajar T, Jallila O, Hind A, Mohammed S, Nadia G. Exploring ethical dilemmas in pediatric oncology nursing: A qualitative approach. *Nursing ethics*. 2026:1. doi:10.1177/09697330261455496

42.	Hasanpour M, Salehpoor-Emran M, Kenner C, Banazadeh M. The resuscitation team members' lived experiences regarding futile cardiopulmonary resuscitation in children and newborn infants: A phenomenological study. *Scientific Reports*. 2026;16(1). doi:10.1038/s41598-026-62073-6

43.	Ishola KE, Oyediran OO, Ayandiran EO, Faremi FA. The Influence of the Yoruba Culture on the Lived Experiences of Clinical Nurses following Death of Patients: A Qualitative Study from a Developing Country. *International Journal of Caring Sciences*. 2024;17(2):1123-32.

44.	Jaaniste T, Helyar M, Eamens M, Smeal T, Coombs S, Mherekumombe M. Challenges, Benefits, and Future Directions of Pediatric Home-Based End-of-Life Care: A Qualitative Study. *Journal of palliative care*. 2026;41(2):127-38. doi:10.1177/08258597251341039

45.	Jongaramraung J, Chanprasit C, Mesukko J, Niyomkar S, Reungrongrat S. End-of-life Decisions for Children in a Thai Pediatric Intensive Care Unit: A Qualitative Descriptive Study. *Pacific Rim International Journal of Nursing Research*. 2020;24(3):321-34.

46.	Kariuki N, Mavumba RWN, Marangu D. KNOWLEDGE, ATTITUDE, PRACTICE, AND BARRIERS TO SERVICE DELIVERY OF PAEDIATRIC PALLIATIVE CARE AMONGST HEALTH CARE PRACTITIONERS IN KENYA – A MIXED METHODS OBSERVATIONAL STUDY. *East African Medical Journal*. 2024;101(3):6818-27.

47.	Kaye EC, Gattas M, Kiefer A, Reynolds J, Zalud K, Li C, et al. Provision of Palliative and Hospice Care to Children in the Community: A Population Study of Hospice Nurses. *Journal of pain and symptom management*. 2019;57(2):241-50. doi:10.1016/j.jpainsymman.2018.10.509

48.	Lee HJ, Kim H. Nurses' perceptions of end-of-life care in neonatal and pediatric intensive care units in Korea: a qualitative descriptive study using thematic analysis. *Child Health Nurs Res*. 2025;31(4):272-83. doi:10.4094/chnr.2025.018

49.	Lima L, Gonçalves S, Pinto C. Sudden death in paediatrics as a traumatic experience for critical care nurses. *Nursing in critical care*. 2018;23(1):42-7. doi:10.1111/nicc.12326

50.	Liu Y, Yang J, Song L, Yang X, Yin Y, Yan L. Nurses' experiences and attitudes toward diagnosis disclosure for cancer patients in China: A qualitative study. *Psycho-oncology*. 2019;28(12):2415-21. doi:10.1002/pon.5273

51.	Lopes VF, Silva JLL, Andrade M. The professionals nursing perception about palliative cares to oncologic pediatric is of cure possibility patient: a study in phenomenological boarding of the relations human beings. *Online Brazilian Journal of Nursing*. 2007;6(3):27-. doi:10.5935/1676-4285.20071155

52.	McAndrew NS, Leske JS. A Balancing Act: Experiences of Nurses and Physicians When Making End-of-Life Decisions in Intensive Care Units. *Clinical nursing research*. 2015;24(4):357-74. doi:10.1177/1054773814533791

53.	Murphy JM, Chin ED, Westlake CA, Asselin M, Brisbois MD. Pediatric Hematology/Oncology Nurse Spirituality, Stress, Coping, Spiritual Well-being, and Intent to Leave: A Mixed-method Study. *Journal of Pediatric Oncology Nursing*. 2021;38(6):349-63. doi:10.1177/10434542211011061

54.	Navarro Silva I, Rejane Salim N, Szylit R, Silva Sampaio PS, de Faria Ichikawa CR, dos Santos MR. Knowing nursing team care practices in relation to newborns in end-of-life situations. *Anna Nery School Journal of Nursing / Escola Anna Nery Revista de Enfermagem*. 2017;21(4):1-8. doi:10.1590/2177-9465-EAN-2016-0369

55.	Nordgren L, Olsson H. Palliative care in a coronary care unit: a qualitative study of physicians' and nurses' perceptions. *Journal of clinical nursing*. 2004;13(2):185-93. doi:10.1111/j.1365-2702.2004.00816.x

56.	Park MJ, Joung WJ, Chung NR, Chae SM. Neonatal Intensive Care Unit Nurses' End-of-Life Care Experience: Becoming a More Empathetic Professional Caregiver. *Advances in neonatal care : official journal of the National Association of Neonatal Nurses*. 2026;26(4):384-93. doi:10.1097/anc.0000000000001380

57.	Pearson H. Children's cancer nurses' experiences of providing palliative care in the acute hospital setting. *Pediatric Blood and Cancer*. 2012;59(6):1129-30. doi:10.1002/pbc.24295

58.	Porter AS, Zalud K, Applegarth J, Woods C, Gattas M, Rutt E, et al. Community Hospice Nurses' Perspectives on Needs, Preferences, and Challenges Related to Caring for Children With Serious Illness. *JAMA Netw Open*. 2021;4(10):e2127457. doi:10.1001/jamanetworkopen.2021.27457

59.	Puia DM, Lewis L, Beck CT. Experiences of obstetric nurses who are present for a perinatal loss. *J Obstet Gynecol Neonatal Nurs*. 2013;42(3):321-31. doi:10.1111/1552-6909.12040

60.	Quiñonez Mora MA, Henao Castaño ÁM, Fajardo Ramos E. Significado de cuidado al final de la vida en la unidad de cuidado intensivo pediátrico. *Cultura de los Cuidados*. 2020;24(57):72-82. doi:10.14198/cuid.2020.57.07

61.	Rashotte J, Fothergill-Bourbonnais F, Chamberlain M. Pediatric intensive care nurses and their grief experiences: a phenomenological study. *Heart & lung : the journal of critical care*. 1997;26(5):372-86. doi:10.1016/s0147-9563(97)90024-8

62.	Rashotte J, King J, Thomas M, Cragg B. Nurses' moral experience of administering PRN anti-seizure medications in pediatric palliative care. *Can J Nurs Res*. 2011;43(3):58-77.

63.	Rico-Mena P, Güeita-Rodríguez J, Martino-Alba R, Castel-Sánchez M, Palacios-Ceña D. The Emotional Experience of Caring for Children in Pediatric Palliative Care: A Qualitative Study among a Home-Based Interdisciplinary Care Team. *Children (Basel, Switzerland)*. 2023;10(4). doi:10.3390/children10040700

64.	Rowe J, Hawley M, Bloomer MJ, O'Neill J. "It's the fear of the unknown" clinicians' perceptions on paediatric compassionate transport to preferred place of death: A qualitative descriptive study. *Australian Critical Care*. 2026;39(5). doi:10.1016/j.aucc.2026.101680

65.	Rüdisüli K, Schlüer AB. [Areas of conflict in the caring of critically chronic ill children: an interpretative phenomenological study]. *Pflege*. 2025;38(2):93-100. doi:10.1024/1012-5302/a001017

66.	Sadeghi N, Hesami SA, Sadeghi M, Sadeghi S. Nurses' Experiences of Palliative Care in the Neonatal Intensive Care Unit: A Qualitative Study. *Avicenna Journal of Nursing and Midwifery Care*. 2021;29(4):302-13. doi:10.30699/ajnmc.29.4.302

67.	Sahin RS, Sümengen AA, Ercan-Koyuncu I, Tekinyildiz E, Günes E, Savas EH, et al. Under the Shadow of Ethical Dilemmas: A Qualitative Study of Pediatric Oncology Nurses' End-of-Life Care Experiences. *Cancer nursing*. 2026. doi:10.1097/ncc.0000000000001599

68.	Salamon E, Fodor É, Földesi E, Hauser P, Kriván G, Csanádi K, et al. The Impact of Qualification and Hospice Education on Staff Attitudes during Palliative Care in Pediatric Oncology Wards—A National Survey. *Children*. 2024;11(2):178. doi:10.3390/children11020178

69.	Saringer-Siegl S, Eßl-Maurer R, Paal P. Nurses' perspectives on implementing pediatric palliative care: a qualitative participatory study on the emerging role of the advanced practice nurse. *Eur J Pediatr*. 2026;185(3). doi:10.1007/s00431-026-06790-0

70.	Sawin KJ, Montgomery KE, Dupree CY, Haase JE, Phillips CR, Hendricks-Ferguson VL. Oncology Nurse Managers' Perceptions of Palliative Care and End-of-Life Communication. *Journal of pediatric oncology nursing : official journal of the Association of Pediatric Oncology Nurses*. 2019;36(3):178-90. doi:10.1177/1043454219835448

71.	Stober S, Metzing S, Reick S. Initiation and implementation of pediatric palliative care in pediatric oncology: A qualitative interview study. *Pflege*. 2026;39(5):231-8. doi:10.1024/1012-5302/a001094

72.	Taplak AS, Gürol A, Polat S. Nurses' Perceptions of the Palliative Care Needs of Neonates With Multiple Congenital Anomalies. *Journal of Hospice & Palliative Nursing*. 2020;22(2):137-44. doi:10.1097/njh.0000000000000628

73.	Taylor J, Aldridge J. Exploring the rewards and challenges of paediatric palliative care work - a qualitative study of a multi-disciplinary children's hospice care team. *BMC palliative care*. 2017;16(1):73. doi:10.1186/s12904-017-0254-4

74.	Tornøe KA, Danbolt LJ, Kvigne K, Sørlie V. The challenge of consolation: nurses' experiences with spiritual and existential care for the dying-a phenomenological hermeneutical study. *BMC nursing*. 2015;14:62. doi:10.1186/s12912-015-0114-6

75.	Verri ER, Santana Bitencourt NA, da Silva Oliveira JA, dos Santos Júnior R, Silva Marques H, Alves Porto M, et al. NURSING PROFESSIONALS: UNDERSTANDING ABOUT PEDIATRIC PALLIATIVE CARE. *Journal of Nursing UFPE / Revista de Enfermagem UFPE*. 2019;13(1):126-36. doi:10.5205/1981-8963-v13i01a234924p126-136-2019

76.	Yang MH, McIlfatrick S. Intensive care nurses' experiences of caring for dying patients: a phenomenological study. *International journal of palliative nursing*. 2001;7(9):435-41. doi:10.12968/ijpn.2001.7.9.9302

77.	Zamalin D, Reed R, Green R, Drago MJ. Comfort and Conflict in Neonatal End-of-Life Care: A Mixed-Methods Study. *The American journal of hospice & palliative care*. 2026:10499091261483161. doi:10.1177/10499091261483161

78.	Alodhialah AM, Almutairi AA, Almutairi M. Exploring Nurses' Emotional Resilience and Coping Strategies in Palliative and End-of-Life Care Settings in Saudi Arabia: A Qualitative Study. *Healthcare (Basel, Switzerland)*. 2024;12(16). doi:10.3390/healthcare12161647

79.	Anderson NE, Kent B, Owens RG. Experiencing patient death in clinical practice: nurses' recollections of their earliest memorable patient death. *International journal of nursing studies*. 2015;52(3):695-704. doi:10.1016/j.ijnurstu.2014.12.005

80.	Betriana F, Kongsuwan W. Grief reactions and coping strategies of Muslim nurses dealing with death. *Nursing in critical care*. 2020;25(5):277-83. doi:10.1111/nicc.12481

81.	Bloomer MJ, Endacott R, Copnell B, O'Connor M. End of life in neonatal and paediatric intensive care: The complex demands of patient and family care. A qualitative exploratory study. *Intensive Care Medicine*. 2013;39:S225. doi:10.1007/s00134-013-3095-5

82.	Brennan E, Guerin S. “Golden” moments and more in grief talk: Healthcare professionals’ approaches to discussing loss and grief with parents of children with severe neurological impairment. *Palliative medicine*. 2026;40(8):1177-86. doi:10.1177/02692163261446187

83.	Dong F, Zheng R, Chen X, Wang Y, Zhou H, Sun R. Caring for dying cancer patients in the Chinese cultural context: A qualitative study from the perspectives of physicians and nurses. *European journal of oncology nursing : the official journal of European Oncology Nursing Society*. 2016;21:189-96. doi:10.1016/j.ejon.2015.10.003

84.	Durmuş Sarıkahya S, Gelin D, Çınar Özbay S, Kanbay Y. Experiences and Practices of Nurses Providing Palliative and End-of-Life Care to Oncology Patients: A Phenomenological Study. *Florence Nightingale journal of nursing*. 2023;31(Supp1):S22-s30. doi:10.5152/fnjn.2023.23042

85.	Jeffers S. Nurse faculty perceptions of end-of-life education in the clinical setting: a phenomenological perspective. *Nurse education in practice*. 2014;14(5):455-60. doi:10.1016/j.nepr.2014.03.009

86.	Kaplan LJ. Toward a model of caregiver grief: Nurses' experiences of treating dying children. *Omega: Journal of Death and Dying*. 2000;41(3):187-206. doi:10.2190/NGG6-YPAH-40AB-CNX0

87.	Kochen EM, Teunissen SCCM, Boelen PA, Jenken F, De Jonge RR, Grootenhuis MA, et al. Challenges in preloss care to parents facing their child's end-of-life: A qualitative study from the health care professionals' perspective. *Palliative medicine*. 2021;35(1 SUPPL):135. doi:10.1177/02692163211035909

88.	Laronne A, Granek L, Wiener L, Feder-Bubis P, Golan H. Organizational and individual barriers and facilitators to the integration of pediatric palliative care for children: A grounded theory study. *Palliative medicine*. 2021;35(8):1612-24. doi:10.1177/02692163211026171

89.	Maunder Z. The emotional labour of children's nurses caring for life-limited children and young people within community and children's hospice settings in wales. *Archives of Disease in Childhood*. 2016;101:A372. doi:10.1136/archdischild-2016-310863.617

90.	Mohammed S, Savage P, Kevork N, Swami N, Rodin G, Zimmermann C. "I'm going to push this door open. You can close it": A qualitative study of the brokering work of oncology clinic nurses in introducing early palliative care. *Palliative medicine*. 2020;34(2):209-18. doi:10.1177/0269216319883980

91.	Muskat B, Greenblatt A, Anthony S, Beaune L, Hubley P, Newman C, et al. The experiences of physicians, nurses, and social workers providing end-of-life care in a pediatric acute-care hospital. *Death studies*. 2020;44(2):105-16. doi:10.1080/07481187.2018.1526829

92.	Papadatou D, Bellali T, Papazoglou I, Petraki D. Greek nurse and physician grief as a result of caring for children dying of cancer. *Pediatric nursing*. 2002;28(4):345-53.

93.	Pestian T, Ellakula JC, Awtrey E, Carle AC, Winick N, Kanov J, et al. Clinician experiences of organisational compassion in healthcare: a qualitative study. *Bmj Open*. 2026;16(5). doi:10.1136/bmjopen-2025-106948

94.	Sisk BA, Friedrich A, Blazin LJ, Baker JN, Mack JW, DuBois J. Communication in Pediatric Oncology: A Qualitative Study. *Pediatrics*. 2020;146(3). doi:10.1542/peds.2020-1193

95.	Vega-Vega P, González-Rodríguez R, Palma-Torres C, Ahumada-Jarufe E, Mandiola-Bonilla J, Oyarzún-Díaz C, et al. Revealing the Meaning of the Mourning Process of Pediatric Nurses Facing the Death of Cancer Patients. *Aquichan*. 2013;13(1):81-91. doi:10.5294/aqui.2013.13.1.5

96.	Vivian L, Argent A. “We didn't do anything wrong, we tried our best, but they just died...we really tried.” End-of-life decisions in the Paediatric Intensive Care Unit (PICU), Red Cross Children's Hospital (RCCWMH), Cape Town, South Africa. *Pediatric Critical Care Medicine*. 2011;12(3):A157. doi:10.1097/PCC.0b013e3182112e80

97.	Beckstrand RL, Rawle NL, Callister L, Mandleco BL. Pediatric nurses' perceptions of obstacles and supportive behaviors in end-of-life care. *American journal of critical care : an official publication, American Association of Critical-Care Nurses*. 2010;19(6):543-52. doi:10.4037/ajcc2009497

98.	Chin SDN, Paraszczuk AM, Eckardt P, Bressler T. Neonatal Nurses' Perceptions of Palliative Care in the Neonatal Intensive Care Unit. *MCN The American journal of maternal child nursing*. 2021;46(5):250-7. doi:10.1097/nmc.0000000000000738

99.	Dryden-Palmer K, Gregoroff P, Parshuram C. End of life conversations in critical care: A simulation intervention for critical care nurses. *Pediatric Critical Care Medicine*. 2014;15(4):201. doi:10.1097/01.pcc.0000449635.80539.f4

100.	Gijón-Mediavilla M, Canet Tarrés A, Oviedo Melgares L, López Fernández E, Rojo Conejo P, Belda Hofheinz S. Health Professionals' Perceptions of End-of-Life Care in the PICU: Single-Center Survey in Spain. *Pediatric critical care medicine : a journal of the Society of Critical Care Medicine and the World Federation of Pediatric Intensive and Critical Care Societies*. 2026;27(9):1054-62. doi:10.1097/pcc.0000000000003978

101.	González-Astray S, Marrero-González CM, González-Pérez I, Arbelo-Molina J, García Hernández AM, González-Darias A. Experiences of NICU Nurses Facing Perinatal Death: A Phenomenological Study. *Children (Basel, Switzerland)*. 2026;13(6). doi:10.3390/children13060795

102.	Hamric AB, Blackhall LJ. Nurse-physician perspectives on the care of dying patients in intensive care units: collaboration, moral distress, and ethical climate. *Critical care medicine*. 2007;35(2):422-9. doi:10.1097/01.Ccm.0000254722.50608.2d

103.	Jiménez Flores J, Casado Rodríguez A, Fernández Tuñas MC, Láiz Rodríguez ME, Salcedo Madridejos Á, Sánchez Suárez P, et al. First national study on the ethical dimension of neonatal nursing. *Nursing ethics*. 2026. doi:10.1177/09697330261465871

104.	Kalicińska M, Chylińska J, Wilczek-Różyczka E. Professional burnout and social support in the workplace among hospice nurses and midwives in Poland. *International journal of nursing practice*. 2012;18(6):595-603. doi:10.1111/ijn.12003

105.	Reid BB, Brogan P. Nurses' experiences of providing palliative care for children with life-limiting conditions. *International journal of palliative nursing*. 2024;30(5):212-24. doi:10.12968/ijpn.2024.30.5.212

106.	Saati HS. Compassion Fatigue, Satisfaction and Burnout AmongOncology Nurses Working in Pediatric Oncology Setting. *Bioscience Biotechnology Research Ccommunications*. 2020;13(3):1314-20. doi:/10.21786/bbrc/13.3/50

107.	Almeida Fde A, Moraes MS, Cunha ML. Taking care of the newborn dying and their families: Nurses' experiences of neonatal intensive care. *Revista da Escola de Enfermagem da U S P*. 2016;50 Spec:122-9. doi:10.1590/s0080-623420160000300018

108.	Bergsträsser E, Cignacco E, Luck P. Health care Professionals' Experiences and Needs When Delivering End-of-Life Care to Children: A Qualitative Study. *Palliative care*. 2017;10:1178224217724770. doi:10.1177/1178224217724770

109.	Broden Arciprete EG, Eche-Ugwu IJ, Decourcey DD, Rosenberg AR, Snaman JM. “Leave No Question Unanswered”: Nurses’ Role in Preparing Children’s Families During End-of-Life Care. *American Journal of Critical Care*. 2025;34(5):380-4. doi:10.4037/ajcc2025791

110.	Broden EG, Eche-Ugwu IJ, DeCourcey DD, Wolfe J, Hinds PS, Snaman J. "At Least I Can Push this Morphine": PICU Nurses' Approaches to Suffering Among Dying Children. *Journal of pain and symptom management*. 2024;68(2):132-41.e2. doi:10.1016/j.jpainsymman.2024.04.018

111.	de Abreu Ferro T, da Silva LTP, Silva-Rodrigues FM, dos Santos MR, Szylit R. Nurses’ beliefs in the care of newborns at the end of life in the neonatal intensive care unit. *Revista da Escola de Enfermagem da USP*. 2024;58. doi:10.1590/1980-220X-REEUSP-2024-0065PT

112.	Dönmez Ç, Kara R, Muslu K, Johnston B. Nurses' experiences of end-of-life communication with children who have life-threatening conditions in paediatric intensive care units: a qualitative descriptive study. *BMC palliative care*. 2026;25(1). doi:10.1186/s12904-026-02007-1

113.	Ferro TD, da Silva LTP, Silva-Rodrigues FM, dos Santos MR, Szylit R. Nurses' beliefs in the care of newborns at the end of life in the neonatal intensive care unit. *Revista Da Escola De Enfermagem Da Usp*. 2024;58. doi:10.1590/1980-220X-REEUSP-2024-0065en

114.	Forster E, Hafiz A. Paediatric death and dying: exploring coping strategies of health professionals and perceptions of support provision. *International journal of palliative nursing*. 2015;21(6):294-301. doi:10.12968/ijpn.2015.21.6.294

115.	Ghaljeh M, Rezaee N, Mardani-Hamooleh M. Nurses' effort for providing end-of-life care in paediatric oncology: a phenomenological study. *International journal of palliative nursing*. 2023;29(4):188-95. doi:10.12968/ijpn.2023.29.4.188

116.	Groves KA, Adewumi A, Gerhardt CA, Skeens MA, Suttle ML. Grief in critical care nurses after pediatric suffering and death. *Annals of palliative medicine*. 2022;11(6):1888-99. doi:10.21037/apm-21-3225

117.	Han S, Min H, Kim S. NICU nurses' moral distress surrounding the deaths of infants. *Nursing ethics*. 2023;30(2):276-87. doi:10.1177/09697330221134978

118.	Hjorth E, Melin-Johansson C, Holmberg B, Godskesen T, Hagelin CL, Ozanne A, et al. Navigating existential conversations about life and death with families in paediatric palliative care: a qualitative interview study with healthcare professionals. *BMC palliative care*. 2026;25(1). doi:10.1186/s12904-026-02171-4

119.	Hyun-Ju K, Kyung-Sook B. Neonatal Intensive Care Unit Nurses' Experience in Caring for Infants Who Are Dying. *Child Health Nursing Research*. 2013;19(4):252-61. doi:10.4094/chnr.2013.19.4.252

120.	Kain VJ. An exploration of the grief experiences of neonatal nurses: A focus group study. *Journal of Neonatal Nursing*. 2013;19(2):80-8. doi:10.1016/j.jnn.2012.04.001

121.	Lin C-H, Liu Y-C, Chiang H-H. From self‐compassion to compassionate action: Reflecting on ending life of stillbirth care in nursing. *Scandinavian Journal of Caring Sciences*. 2021;35(1):260-7. doi:10.1111/scs.12842

122.	Lu C-W, Wong T-T, Mu P-F. Providing end-of-life nursing care to children with cancer. *Journal of Nursing*. 2011;58(4):37-46.

123.	Montgomery KE, Sawin KJ, Hendricks-Ferguson V. Communication During Palliative Care and End of Life. *Cancer nursing*. 2017;40(2):E47-E57. doi:10.1097/NCC.0000000000000363

124.	Oguzhan H, Akay G, Uzun S, Koç ES, Öncer A. Surrogate Trauma in Nurses Providing Pediatric Palliative Care: A Phenomenological Study. *Journal of Hospice & Palliative Nursing*. 2026;28(4):E178-E85. doi:10.1097/njh.0000000000001209

125.	Oliveira F, Cleveland LM, Darilek U, Borges Silva AR, Carmona EV. Brazilian Neonatal Nurses' Palliative Care Experiences. *J Perinat Neonatal Nurs*. 2018;32(4):E3-e10. doi:10.1097/jpn.0000000000000361

126.	Oyediran OO, Ishola KE, Ayandiran EO, Olatubi IM. Nurses' Lived Experiences Following End-of-Life Care: A Hermeneutic Study from a North Central State, Nigeria. *Nurse Media Journal of Nursing*. 2025;15(2):123-36. doi:10.14710/nmjn.v15i2.63330

127.	Papadatou D, Martinson IM, Chung PM. Caring for dying children: a comparative study of nurses' experiences in Greece and Hong Kong. *Cancer nursing*. 2001;24(5):402-12. doi:10.1097/00002820-200110000-00013

128.	Peterson J, Johnson M, Halvorsen B, Apmann L, Chang PC, Kershek S, et al. Where do nurses go for help? A qualitative study of coping with death and dying. *International journal of palliative nursing*. 2010;16(9):432, 4-8. doi:10.12968/ijpn.2010.16.9.78636

129.	Sadeghi N, Hossein Hesami SA, Sadeghi S, Sadeghi M. Barriers to Palliative Care in the Neonatal Intensive Care Unit from Nurses’ Perspective: A Qualitative Study. *Medical-Surgical Nursing Journal*. 2021;10(2):1-8. doi:10.5812/msnj.120664

130.	Suo TH, Lu Q, Rui XL, Yang GQ, Wang LP, Wang L. Chinese Neonatal Nurses' Lived Experiences of Providing End-of-Life Care in the NICU. *Advances in Neonatal Care*. 2023;23(6):532-40. doi:10.1097/anc.0000000000001113

131.	Suryani RL, Allenidekania A, Rachmawati IN. Phenomenology Study on Nurses' Experiences in Understanding the Comfort of Children at the End-of-Life. *Indian journal of palliative care*. 2018;24(2):162-6. doi:10.4103/ijpc.Ijpc_200_17

132.	Teixeira GDFA, Batista PSdS, Lima DRAd, Oliveira AMdM, Dias KCCDO, Costa BHS. Palliative Care in Oncology: Nurses’ Experience in Caring for Children in The Final Stages of Life. *Revista de Pesquisa Cuidado é Fundamental Online*. 2020:689-95. doi:10.9789/2175-5361.rpcfo.v12.9463

133.	Tranter S, Josland E, Turner K. NURSES' BEREAVEMENT NEEDS AND ATTITUDES TOWARDS PATIENT DEATH: A QUALITATIVE DESCRIPTIVE STUDY OF NURSES IN A DIALYSIS UNIT. *Journal of renal care*. 2016;42(2):101-6. doi:10.1111/jorc.12147

134.	Yam BM, Rossiter JC, Cheung KY. Caring for dying infants: experiences of neonatal intensive care nurses in Hong Kong. *Journal of clinical nursing*. 2001;10(5):651-9. doi:10.1046/j.1365-2702.2001.00532.x

135.	Zhang M, Wang D. Professional grief experience and psychological detachment of pregnant emergency nurses after the death of pediatric patients: A longitudinal mixed-methods study. *Frontiers in Psychology*. 2026;16. doi:10.3389/fpsyg.2025.1715162

136.	Brimble MJ. HOW DO CHILDREN'S NURSE WORKING IN HOSPICES MANAGE EMOTIONAL LABOUR AND PROFESSIONAL INTEGRITY IN LONG-TERM RELATIONSHIPS WITH PARENTS? *BMJ Supportive and Palliative Care*. 2023;13:A20. doi:10.1136/spcare-2023-PCC.52

137.	Curcio DL. The Lived Experiences of Nurses Caring For Dying Pediatric Patients. 2017.

138.	Du Pré P, Brierley J, Koffman J. The experiences, attitudes and practices of nurses working in a paediatric intensive care unit caring for babies and children at the end of life: A qualitative study. *Archives of Disease in Childhood*. 2018;103:A196. doi:10.1136/archdischild-2018-rcpch.468

139.	Kline N, Thom B. The lived experience of grief, loss, and coping among pediatric oncology nurses (733). *Journal of pain and symptom management*. 2011;41(1):292. doi:10.1016/j.jpainsymman.2010.10.212

140.	Porter A, Reeves T, Williams K, Baker J, Kaye E. “On the Other Side of the Fence”: Impact of Personal Experiences on Career Path, Clinical Practice, and Professional Endurance Among Hospice Nurses Caring for Dying Children (S559). *Journal of pain and symptom management*. 2022;63(5):938-9. doi:10.1016/j.jpainsymman.2022.02.182

# **Supplementary Table 2.** Characteristics of the included studies (n = 37)

| **Authors, year, country** | **Study aims** | **Study design** | **Setting** | **Participant characteristics** | **Sample size** | **Sampling** | **Data collection** | **Data analysis** |
| --- | --- | --- | --- | --- | --- | --- | --- | --- |
| Abbasi, 2026, Iran | To explore coping strategies employed by nurses in response to patient deaths in pediatric oncology units. | Descriptive qualitative study | Pediatric oncology departments in two referral hospitals in Iran. | Group: pediatric oncology nurses who had experienced pediatric patient death.  Sex: female n=20  Age: range 25-56 years; 25-34 years n=3; 35-44 years n=10; 45-54 years n=7  Education: bachelor’s degree n=19; master’s degree n=1  Nursing experience: range 3-30 years; <=10 years n=3; 11-20 years n=14; >20 years n=3  Specialty/PC experience: pediatric oncology experience range 2-29 years; <=10 years n=8; 11-20 years n=10; >20 years n=2  PC/EOL training: NR | 20 nurses | Purposive sampling via head-nurse consultation and eligibility review | Unstructured and in-depth semi-structured interviews | Conventional content analysis |
| Alm, 2026, Denmark | To ask what it is like for nurses to accompany children and families through illness, dying and bereavement in home-based paediatric palliative care. | Phenomenological-hermeneutical study | Regional home-based paediatric palliative care teams in Denmark | Group: nurses providing home-based pediatric palliative care.  Sex: female n=10  Age: 37-65 years  Education: NR  Nursing experience: 11-41 years; mean 28.2 years  Specialty/PC experience: PPC team experience 1-9 years; some also worked in neonatal units, pediatric oncology or intensive care  PC/EOL training: NR | 10 nurses | Total population sampling within Danish PPC teams | Phenomenological individual interviews | Van Manen thematic reflection |
| Anguis, 2023, Spain | To explore the feelings, experiences, perceptions, and coping strategies of pediatric ICU nurses after experiencing the death of a child. | Qualitative exploratory study based on Van Manen’s hermeneutic phenomenology | Level III Pediatric Intensive Care Unit (PICU) of a public referral hospital in southeast Spain | Group: PICU nurses who had directly cared for critically ill children who died in the PICU.  Sex: 9 female, 1 male  Age: 22–47 years (median 36 years)  Education: Pediatric nurse specialists or pediatric nurse trainees (Spain's pediatric nursing specialty program)  Nursing experience: 2–25 years (median 14 years)  PICU experience: 1–22 years (median 13 years)  Specialty/PC experience: All had direct experience caring for children who died in the PICU; ≥1 year PICU experience required; 30% had previous adult ICU experience.  PC/EOL training: NR | 10 nurses | Snowball convenience sampling with key-informant recruitment | Semi-structured individual interviews | Latent content analysis using a priori interview-topic codes and emergent codes, within a hermeneutic phenomenological framework. |
| Bassola, 2023, Italy | To understand nurses' experiences of caring for infants and children with life-limiting and life-threatening conditions | Qualitative, phenomenological descriptive study | Pediatric ward and neonatal intensive care unit in a general hospital in Northern Italy | Group: nurses working in pediatric and neonatal care settings.  Sex: female n=25; male n=2  Age: mean 43.7 years; range 26–58 years  Education: NR; professional qualification: general nurse n=16, pediatric nurse n=11  Nursing experience: total nursing experience NR  Specialty/PC experience: years in the operating unit, mean 18.2 (range 1–38); pediatric ward n=12; NICU n=15  PC/EOL training: NR | 27 nurses | Purposive sampling | In-depth interviews | Colaizzi phenomenological analysis |
| Bian, 2023, China | To explore pediatric nurses' challenges and effective coping strategies in caring for dying children | Descriptive qualitative study | Grade 3A hospital in Hanzhong City, Shaanxi Province, China | Group: pediatric nurses with experience caring for dying children.  Sex: female n=10  Age: 25-49 years; mean 35.5 years  Education: college degree n=6; undergraduate degree n=4  Nursing experience: more than 1 year in hospital eligible  Specialty/PC experience: experience caring for dying children in the past 5 years  PC/EOL training: NR | 10 nurses | Maximum variation purposive sampling via recruitment poster and WeChat notice | Semi-structured interviews | Krippendorff content analysis |
| Bloomer, 2015, Australia | To explore how NICU/PICU nurses care for families before and after the death of an infant or child and the emotional content and sources of discomfort in that care | Mixed methods study | NICU and PICU settings in two major Australian metropolitan hospitals | Group: registered nurses working in NICU/PICU with at least 1 year of experience.  Sex: NR  Age: NR  Education: NR  Nursing experience: at least 1 year in NICU/PICU eligible  PC/EOL experience: NICU n=9; PICU n=13; experience caring for at least one child/infant who had died in the NICU/PICU  PC/EOL training: NR | 22 nurses | Self-selection sampling via manager-mediated invitation | Focus groups and individual interviews | Inductive content analysis; LIWC emotional content analysis |
| Briere, 2025, USA | To understand the role of experienced and morally resilient pediatric intensive care registered nurses and nurse practitioners as they guide families from curative to comfort care. | Qualitative descriptive study. | Four neonatal/pediatric intensive care units at Boston Children's Hospital, United States. | Group: registered nurses and nurse practitioners working in neonatal/pediatric intensive care units.  Sex: female n=19; male n=1  Age: 26-35 years n=6; 36-45 years n=7; 46-55 years n=2; 56-60 years n=5  Education: PhD n=1; DNP n=2; master’s degree n=11; bachelor’s degree n=5; diploma n=1  Nursing experience: current unit mean 15.13 years; median 13.50 years; range 2.5-36 years  Specialty/PC experience: primary unit NICU n=9, MICU n=3, MSICU n=3, CICU n=5; current direct-care role >=2 years and curative-to-comfort transition experience in previous 48 months eligible  PC/EOL training: palliative care training reported among n=17 (on-the-job n=11; formal program n=3; other n=1; not applicable n=4); EOL care training reported among n=18 (on-the-job n=15; formal program n=1; other n=2; not applicable n=1)  Training items allowed multiple responses; category totals are not mutually exclusive. | 20 participants: 7 registered nurses and 13 nurse practitioners. | Peer/leader-nominated purposive sampling with RMRS screening | Zoom interviews | Qualitative thematic analysis |
| Brimble, 2024, UK | To explore emotional labour in children's hospice nursing and how nurses balance professional boundaries, personability and coping. | Narrative interpretive approach study. | Children’s hospice respite/day care across the United Kingdom | Group: Children's hospice nurses.  Sex: female n=5; male n=1  Age: 35-40 years n=1; 41-45 years n=2; 46-50 years n=1; 51-60 years n=2  Education: NR  Nursing experience: 15-34 years  Specialty/PC experience: children's hospice experience 4-32 years; minimum 4 years eligible  PC/EOL training: NR | 6 nurses | Purposive sampling | Audio diaries followed by telephone interviews | Paradigmatic narrative inquiry with inductive thematic analysis |
| Budak, 2025, Turkey | To explore the end-of-life care experiences of pediatric nurses in Turkey | Phenomenological study | Six hospitals of three types (university, private and public) across eight pediatric units and four cities in Turkey | Group: pediatric nurses working in ICU, NICU, pediatric wards and related pediatric specialty units.  Sex: female n=16; male n=2  Age: 25–45 years  Education: bachelor’s degree n=10; master’s degree n=5; high school n=3  Nursing experience: 1–23 years  Specialty/PC experience: pediatric setting experience 6 months–18 years across PICU, NICU, oncology, hematology/oncology and wards  PC/EOL training: NR | 18 nurses | Purposive and snowball sampling | In-depth semi-structured interviews (face-to-face or Zoom). | Braun and Clarke thematic analysis |
| Chew, 2021, Singapore | To explore the experiences, challenges and coping strategies of new nurses dealing with pediatric death in a clinical setting | Descriptive qualitative study | A tertiary public hospital; the largest women's and children's health-care hospital in Singapore | Group: new nurses working in a pediatric setting.  Sex: female n=11; male n=1  Age: 21-24 years n=1; 25-30 years n=9; >30 years n=2  Education: bachelor's degree n=7; diploma n=5  Nursing experience: pediatric setting <1 year n=1; 1-2 years n=8; 2-3 years n=3; less than 3 years in pediatric ward setting eligible  Specialty/PC experience: critical care n=7; high dependency n=2; oncology ward n=3  PC/EOL training: one participant had recently undergone palliative training; most reported limited death/palliative-care preparation. | 12 nurses | Purposive sampling | Semi-structured interviews | Thematic analysis |
| Chong, 2017, Malaysia | To explore the experience of community palliative care nurses providing home care to children. | Qualitative study | Three community palliative care provider organizations in greater Kuala Lumpur, Malaysia | Group: community palliative care nurses providing home care to children.  Sex: all female  Age: median 29.5 years; range 24-62 years  Education: NR  Nursing experience: median 5 years; range 1-25 years  Specialty/PC experience: palliative care nurse experience median 2.5 years; range 1-18 years; only 1 had prior pediatric experience  PC/EOL training: none had pediatric palliative care training | 16 nurses | Total population sampling within community palliative care organizations | Semi-structured in-depth interviews | Thematic analysis |
| Cook, 2012, USA | To describe and understand behavior and coping strategies used by pediatric nurses caring for dying children on an inpatient acute care cardiology unit. | Qualitative descriptive study | 41-bed inpatient acute cardiology unit in a freestanding children's academic tertiary-care center in the United States | Group: acute care nurses and pediatric nurse practitioners.  Sex: NR  Age: NR  Education: NR  Nursing experience: 1-5 years n=7; 6-10 years n=5; >=11 years n=10; range 1-33 years  Specialty/PC experience: staff nurses n=17; pediatric nurse practitioners n=5; staff nurse level I n=6, level II n=7, level III n=4; all had cared for at least one acutely ill child who died in the hospital  PC/EOL training: NR | 22 nurses/nurse practitioners | Criterion-based convenience sampling via unit-wide e-mail invitation | Three focus groups | Conventional content analysis |
| Devitt, 2021, Ireland | To identify whether nurses in the acute setting face problems when caring for children with life-limiting conditions and to highlight what may be unique to a general paediatric ward. | Qualitative study | Paediatric wards in a general university hospital in Ireland | Group: registered nurses working in the acute paediatric unit and caring for children with life-limiting conditions.  Sex: all female  Age: 26-54 years  Education: NR  Nursing experience: 1 to >20 years  Specialty/PC experience: experience caring for children with life-limiting conditions in acute paediatric settings  PC/EOL training: many reported inefficient or limited PPC education/training. | 14 nurses | Purposive convenience sampling | Semi-structured interviews | Braun and Clarke thematic analysis |
| Ebadinejad, 2022, Iran | To explore the palliative care strategies of Iranian nurses for children dying from cancer. | Conventional qualitative content analysis study | Paediatric cancer ward of a reference hospital in Semnan, Iran | Group: nurses caring for children with cancer.  Sex: NR  Age: NR  Education: NR  Nursing experience: pediatric oncology nursing experience >=6 months eligible; maximum variation in work experience considered  Specialty/PC experience: 8 nurses in a 16-participant mixed sample; nurses with longest pediatric oncology experience selected as key informants  PC/EOL training: NR | 16 participants overall, including 8 nurses (nursing data extracted for this review) | Maximum variation purposive sampling | In-depth individual unstructured and semi-structured interviews | Graneheim and Lundman content analysis |
| Elsharkawy, 2025, Saudi Arabia | To explore and describe the lived experience of resilience and coping strategies among neonatal intensive care nurses following infant loss in NICUs in northern Saudi Arabia | Interpretive hermeneutic phenomenological study | Four NICUs in northern Saudi Arabia: three public tertiary care centers and one private hospital | Group: registered NICU nurses exposed to infant loss  Sex: NR  Age: 25-49 years  Education: diploma n=4; bachelor's degree n=11; master's degree n=3  Nursing experience: NR  Specialty/PC experience: NICU experience 2–18 years; ≥2 years NICU experience eligible; cared for at least three infants who subsequently died; neonatal loss within preceding 24 months eligible  PC/EOL training: NR | 18 nurses | Maximum variation purposive sampling via nursing-director dissemination. | Semi-structured interviews, reflective journals and follow-up interviews | Interpretive phenomenological analysis |
| Epstein, 2008 and 2010, USA | To explore nurses’ and physicians’ end-of-life experiences in the newborn intensive care unit. & To explore nurses’ and physicians’ moral obligations in providing neonatal end-of-life care. | Hermeneutic phenomenological qualitative study | 45-bed level III NICU in a mid-Atlantic university hospital serving a large rural area in the United States | Group: NICU nurses, nurse practitioners and physicians who provided end-of-life care; nursing findings extracted for this review  Sex: female n=18; male n=1 among nurses with returned questionnaires  Age: mean 36.2 years; range 25-57 years  Education: diploma n=3; associate degree n=7; bachelor’s degree n=6; master’s degree n=2; unreported n=1  Nursing experience: mean 12.6 years; range 1-28 years  Specialty/PC experience: NICU nurses/nurse practitioners providing EOL care; 21 nurses interviewed, 19 returned demographic questionnaires  PC/EOL training: formal EOL education yes n=5; no n=14 | 21 nurses and 11 physicians | Purposive sampling | Semi-structured interviews | Epstein 2008: Hermeneutic circle analysis of neonatal end-of-life experiences; Epstein 2010: Conventional content analysis of moral obligations |
| Erikson, 2017, USA | To explore how nurses manage personal and professional boundaries in caring for seriously ill children and families. | Constructivist grounded theory study. | Freestanding pediatric palliative care center and academic tertiary-care children's hospital units in a large Western US metropolitan area. | Group: registered nurses in a PPC center, oncology unit, intensive care nursery and PICU.  Sex: female n=16; male n=2  Age: mean 39.2 years; range 25-59 years  Education: diploma n=1; associate degree n=2; bachelor's degree n=10; master's degree n=5  Nursing experience: mean 15.69 years; range 2.5-44.0 years  Specialty/PC experience: years in work setting mean 3.79; pediatric experience mean 12.75 years; PPC center n=10; children's hospital n=8  PC/EOL training: NR | 18 registered nurses | Purposeful and snowball sampling. | Semi-structured interviews. | Constructivist grounded theory analysis / constant comparative analysis. |
| Gürcan, 2025, Turkey | To develop an explanatory framework to examine pediatric oncology nurses' experiences and emotional challenges in providing end-of-life care | Grounded theory study | Tertiary university hospital with two specialized pediatric hematology and oncology clinics in Turkey | Group: pediatric oncology nurses providing end-of-life care  Sex: female n=14  Age: mean 32.2 years; range 25-43 years  Education: undergraduate n=12; master's degree n=2  Nursing experience: professional experience 2-20 years  Specialty/PC experience: pediatric oncology clinic experience 1-10 years  PC/EOL training: NR | 14 nurses | Purposive and theoretical sampling | Face-to-face and online semi-structured interviews with researcher field notes | Grounded theory analysis with constant comparative analysis |
| Henao-Castaño, 2019, Colombia | To explore nurses’ experiences of coping with death in a pediatric intensive care unit. | Hermeneutic phenomenological study. | PICU of the Meintegral Health Service Provider Institution in Manizales, Colombia. | Group: nurses working in a pediatric intensive care unit.  Sex: female n=8; male n=2  Age: 26-38 years  Education: NR  Nursing experience: more than 1 year in PICU eligible  Specialty/PC experience: experience of the death of a paediatric patient eligible  PC/EOL training: NR | 10 nurses | Convenience sampling. | Semi-structured in-depth interviews. | Cohen, Kahn and Steeves phenomenological analysis. |
| Hendricks-Ferguson, 2015, USA | To describe commonalities of novice pediatric oncology nurses’ experiences with palliative care and end-of-life communication and factors that facilitate or impede communication. | Qualitative empirical phenomenology study. | Three major pediatric hospitals in the Midwest region of the United States. | Group: novice registered nurses working in pediatric oncology settings.  Sex: all Caucasian female  Age: 25-31 years; mean 28 years  Education: associate degree n=9; bachelor's degree n=5  Nursing experience: <1 year in pediatric oncology  Specialty/PC experience: experience caring for at least one child with cancer who had died  PC/EOL training: none had attended an educational program focused on PC/EOL care for children or families | 14 nurses | Homogeneous purposive sampling with nurse-manager-assisted recruitment. | Focus group discussions. | Adapted Colaizzi phenomenological analysis. |
| Jucker, 2024, Switzerland | To gain a more comprehensive understanding of specialized nurses’ attitudes toward the use and purpose of ECMO in PICU/NICU and their ethical concerns | Descriptive qualitative study | Mixed NICU/PICU ECMO center in a Swiss University Children's Hospital | Group: critical care nurses with at least 2 years of ECMO experience in a NICU/PICU center  Sex: female n=20; male n=1  Age: mean 36.0 years; range 26-52 years  Education: NR  Nursing experience: PICU/NICU experience mean 10.0 years; range 2-25 years  Specialty/PC experience: critical care nurses with at least 2 years of ECMO experience in NICU/PICU  PC/EOL training: NR | 21 nurses | Purposive sampling | Focus groups | Reflexive thematic analysis |
| Lewis, 2017, USA | To explore, through lived and told stories, the affective, interactional, and meaning-related responses that NICU nurses have while caring for dying infants and their families | Qualitative narrative study | NICU nurses recruited through the National Association of Neonatal Nurses online membership in the United States | Group: NICU registered nurses who were current NANN members  Sex: female n=36  Age: mean 49.7 years; 25-35 n=6; 36-45 n=4; 46-55 n=11; 56-65 n=15  Education: diploma n=1; associate degree n=1; bachelor’s degree n=11; master’s degree n=15; MSN/nurse practitioner n=5; PhD/nurse practitioner n=2  Nursing experience: NICU experience mean 23 years; 0–10 n=7; 11–25 n=12; 26–35 n=11; 36–45 n=5  Specialty/PC experience: level II NICU n=2; level III n=21; level IV n=12  PC/EOL training: EOLC education in school yes n=20; no n=13 | 36 nurses | Purposive online recruitment through NANN membership | Written narratives | Riessman’s thematic narrative analysis |
| Ma, 2021, China | To explore the cognitive, emotional, and behavioral aspects of pediatric oncology nurses’ experiences of patient deaths. | Descriptive qualitative study | Pediatric oncology wards in three children's hospitals in Jiangsu Province, China | Group: pediatric oncology ward nurses who had experienced the death of child patients  Sex: female n=18; male n=4  Age: 25-39 years  Education: junior college n=12; university n=10  Nursing experience: professional years 2-19 years  Specialty/PC experience: pediatric oncology ward nurses who had experienced child patient death within the past year  PC/EOL training: lack of death/grief education and continuing training emphasized; individual training NR | 22 nurses | Purposive sampling via nursing-manager recommendation | Face-to-face semi-structured interviews | Braun and Clarke thematic analysis |
| MacDermott 2014 & Keenan, 2016, Ireland | To explore the personal grief experience of nurses who have cared for a child with an intellectual disability who has died, with a view to drawing awareness to this area, to potentially enhance the body of knowledge, and to develop practice to improve this area of care and support | Qualitative descriptive study | Respite, residential and school environments within an Irish health-care organization for people with intellectual disability | Group: registered nurses who had worked with a child with intellectual disability who died  Sex: female n=8  Age: 21-30 years n=2; 31-40 years n=3; 41-50 years n=1; 61-70 years n=2  Education: nursing registration type reported as intellectual disability n=5; general n=3  Nursing experience: 5-10 years n=2; 11-15 years n=3; 16-20 years n=1; 36-40 years n=1; >=41 years n=1  Specialty/PC experience: registered nurses caring for children with intellectual disability who had died; all had experienced >2 deaths  PC/EOL training: palliative training n=4; pediatric training n=3; intellectual disability training n=6; bereavement training n=0 | 8 nurses | Purposive sampling via gatekeeper-facilitated invitation. | Semi-structured interviews. | Pragmatic qualitative data analysis with open coding. |
| McCloskey, 2010, UK | To explore occupational stress in nurses providing palliative care to children and young people in Northern Ireland | Qualitative focus-group study | Children's hospice, community children's nursing teams and regional children's hospital specialist services across Northern Ireland | Group: children's palliative care nurses from hospice, community and hospital specialist settings  Sex: NR  Age: NR  Education: NR  Nursing experience: identifiable demographic information not reported to protect anonymity  Specialty/PC experience: 18 children's palliative care nurses: hospice n=9; community children's nursing teams n=7; hospital specialist nurses n=2  PC/EOL training: nurses had contributed to care of young people requiring end-of-life care in previous 12 months; individual training NR | 18 nurses | Purposeful sampling from regional children’s palliative care nurse services | Four focus group discussions | Newell and Burnard thematic content analysis |
| Nugent, 2022, Ireland | To explore the experiences of children's nurses who work with seriously ill children and to gain an insight into the dynamics involved in working with children and their families, as well as the nurses' experiences of managing their own vulnerability | Qualitative study with interpretive phenomenological analysis | Large tertiary children's hospital in Dublin, Republic of Ireland | Group: children's nurses working directly with seriously ill children and families  Sex: NR  Age: 21-29 years n=1; 40-49 years n=4  Education: NR  Nursing experience: 7-30 years  Specialty/PC experience: children's nurses who had worked with seriously ill children for at least 5 years; clinical nurse specialists n=2 and staff nurses n=3  PC/EOL training: NR | 5 nurses | Purposive sampling | In-depth semi-structured interviews | Interpretive phenomenological analysis |
| Özsavran, 2026, Turkey | To determine how nurses working in a pediatric intensive care unit remembered and made sense of their experiences regarding children's deaths, an issue with considerable and lasting effects | Descriptive qualitative study involving an interpretative phenomenological analysis approach | Pediatric intensive care unit of a university hospital in the Western Black Sea Region of Turkey | Group: Nurses working in a pediatric intensive care unit  Sex: female n=9; male n=4  Age: 24-39 years  Education: associate degree n=2; undergraduate degree n=10; postgraduate degree n=1  Nursing experience: 2-14 years  Specialty/PC experience: PICU experience 2–14 years; had cared for/witnessed dying child patients eligible  PC/EOL training: NR | 13 nurses | Purposive sampling | Semi-structured interviews | Content analysis |
| Pant, 2026, UK | To explore NICU healthcare personnel's lived experiences of newborn death and supporting grieving and bereaved parents. | Hermeneutic phenomenological study. | Three tertiary NICUs in the United Kingdom. | Group: healthcare personnel in tertiary NICUs, including nurses.  Sex: female n=8 in total mixed healthcare personnel sample  Age: NR  Education: NR  Nursing experience: nurse subgroup NICU experience 7-20 years  Specialty/PC experience: five nurses and three doctors in an eight-participant tertiary NICU healthcare personnel sample; specific ethnicity, background and hospital details not shared to maintain anonymity  PC/EOL training: NR | 8 healthcare personnel, including 5 nurses | Purposive sampling through NICU team introduction sessions and e-mail invitations | Online semi-structured in-depth interviews via Microsoft Teams | Van Manen’s hermeneutic phenomenological analysis |
| Papadatou, 1999, Greece | To explore the sources, nature, and effectiveness of mutual support among nurses who are exposed to the death of children and to compare the experiences between a group of pediatric oncology nurses and a group of critical care nurses. | Qualitative analysis of mutual support within a larger grounded theory study of professional grief. | Pediatric oncology unit and pediatric intensive care unit of Children’s Hospital A. & P. Kyriakou, a large public pediatric hospital in Athens, Greece. | Group: Pediatric oncology nurses and pediatric critical care nurses. Sex: female n=34; male n=5 Age: 21-30 years n=10; 31-40 years n=23; 41-50 years n=6 Education: two-year nursing education n=13; three-year n=22; four-year university n=4 Nursing experience: mean total healthcare experience 10.8 years Specialty/EOL experience: pediatric oncology nurses n=16; pediatric critical care nurses n=23; mean unit experience 7.1 years EOL/PPC training: NR | 16 pediatric oncology nurses and 23 critical care nurses. | Criterion purposive sampling via unit invitation | Semi-structured interviews | Grounded theory-informed qualitative analysis |
| Ramadan, 2025, Egypt | To explore the lived experiences of neonatal palliative care nurses in Egypt, focusing on how they cultivate and sustain compassion in end-of-life care | Qualitative phenomenological study guided by Interpretative Phenomenological Analysis (IPA). | Four NICUs in Gharbia Governorate, Egypt, including public hospitals and academic/teaching medical centers | Group: neonatal palliative care nurses working in NICUs  Sex: female n=36; male n=4  Age: 25-30 years n=12; 31-40 years n=18; 41-55 years n=10  Education: diploma n=8; bachelor’s degree n=24; master’s degree n=8  Nursing experience: 2-5 years n=14; 6-10 years n=16; >10 years n=10  Specialty/PC experience: direct neonatal palliative-care experience; >2 years neonatal care and exposure to palliative cases eligible  PC/EOL training: NR | 40 nurses | Stratified purposive and snowball sampling | In-depth semi-structured interviews (60–90 minutes) and participant reflective journals over two weeks (at least three entries per week). | Interpretative phenomenological analysis |
| Reid, 2013a, Scotland | To elicit the lived experiences of children’s nurses delivering palliative and end of life care to children and young people in the community. | Flexible qualitative design using grounded theory | Rural part of Scotland; children received palliative and end-of-life care at home | Group: community children's nurses and children's respite nurses providing palliative care to children at home.  Sex: all female  Age: NR  Education: trained in the United Kingdom; detailed education NR  Nursing experience: NR  Specialty/PC experience: community children's nurses n=4 and children's respite nurses n=3 providing home palliative/end-of-life care  PC/EOL training: NR | 7 nurses | Indirect targeted purposive sampling via team leads | Single face-to-face semi-structured interviews | Burnard coding, weighting and distillation analysis |
| Reid, 2013b, Scotland | To highlight adult community nurses' experiences of providing palliative care to children and young people in a rural region of Scotland. | Phenomenological study | Rural region of Scotland served by adult community nursing teams from one health-care provider | Group: adult community nurses providing palliative care to children and young people at home.  Sex: NR  Age: NR  Education: community qualifications n=8; palliative care qualifications n=4; children's nursing qualifications n=2  Nursing experience: qualified for 15-42 years; mean 29.5 years  Specialty/PC experience: district nurses n=6 and adult community palliative care clinical nurse specialists n=4; three nurses reported two children/young people cases and seven reported one children/young people case each  PC/EOL training: palliative care qualifications n=4 | 10 nurses | Non-probabilistic recruitment via manager invitation | Semi-structured interviews | Latent inductive content analysis |
| Roach, 2023, Oman | To explore the challenges experienced by nurses providing palliative care to children with leukemia in Oman. | Interpretive phenomenological analysis study | Pediatric hematology-oncology units of a tertiary care hospital in the Sultanate of Oman | Group: registered nurses working in pediatric hematology-oncology units.  Sex: all female  Age: NR  Education: NR  Nursing experience: >1 year caring for children with leukemia eligible; detailed years NR  Specialty/PC experience: registered nurses working in pediatric hematology-oncology units and caring for children with leukemia and families  PC/EOL training: nurses reported need for additional formal and personalized pediatric palliative care training | 11 nurses | Purposive sampling | Semi-structured face-to-face interviews | Interpretative phenomenological analysis |
| Santos, 2014, Brazil | To analyze professional resilience in the life and death process in pediatric care as perceived by health workers | Qualitative study within a larger resilience research project | Two pediatric wards of a mid- and high-complexity hospital in Rio de Janeiro, Brazil | Group: nursing professionals working in pediatric wards  Sex: NR  Age: NR  Education: NR  Nursing experience: 20 nursing professionals interviewed after resilience-scale screening; detailed nursing demographics NR  Specialty/PC experience: nursing professionals working in two pediatric wards caring for children/adolescents with chronic diseases  PC/EOL training: training in life/death care discussed as limited; individual training NR | 20 nursing professionals | Two-stage selection: resilience screening of 56 nursing professionals identified 37 with high scores; 20 were interviewed until saturation. | Semi-structured individual and group interviews | Bardin content analysis guided by symbolic interactionism |
| Shattnawi, 2022, Jordan | To explore Jordanian neonatal intensive care unit nurses' perceptions and needs regarding the provision of EOL care. | Exploratory descriptive qualitative study. | NICUs in two large general hospitals in Irbid, Northern Jordan. | Group: Jordanian NICU nurses.  Sex: female n=12  Age: 25-36 years  Education: bachelor's degree n=9; master's degree n=3  Nursing experience: 3-16 years  Specialty/PC experience: Jordanian NICU nurses with one or more experiences dealing with infant death  PC/EOL training: some had communication-related training; continuing EOL education reported as limited and needed | 12 nurses | Purposive sampling for variation in NICU experience | Semi-structured face-to-face interviews | Thematic analysis |
| Stayer, 2016, USA | To describe and interpret the essence of PICU nurses' experiences providing palliative care to children with life-threatening illnesses and their families | Hermeneutic phenomenological study | PICU of a non-freestanding children's hospital in the northeastern United States | Group: registered nurses working in a pediatric intensive care unit  Sex: female n=9; male n=3  Age: 20-25 years n=2; 26-30 years n=3; 31-40 years n=2; ≥41 years n=5  Education: associate degree n=6; baccalaureate n=6  Nursing experience: 0-5 years n=5; 6-10 years n=3; 11-20 years n=2; >20 years n=2  Specialty/PC experience: PICU practice 0-5 years n=6; 6-10 years n=3; 11-20 years n=1; >20 years n=2  PC/EOL training: formal death/dying education yes n=5; no n=7 | 12 nurses | Purposive sampling | Face-to-face interviews and field notes | Hermeneutic phenomenological analysis |
| Suryani, 2018, Indonesia | To explore and understand the meaning of children's comfort at the end of life for nurses. | Descriptive qualitative phenomenological study | Jakarta home care facility in Indonesia; participants were from Rachel House Foundation | Group: nurses with palliative care training and experience caring for children at end of life.  Sex: NR  Age: NR  Education: palliative-care training certificate required; other education NR  Nursing experience: NR  Specialty/PC experience: nurses with experience caring for children at end of life; recruited from Rachel House Foundation  PC/EOL training: all had attended palliative-care training and held palliative-care training certificates | 10 nurses | Criterion purposive sampling via key-informant nomination and WhatsApp contact | Semi-structured in-depth interviews | Colaizzi phenomenological analysis |

Note: NR = not reported; NICU = neonatal intensive care unit; PICU = pediatric intensive care unit; EOL = end-of-life care; PC = palliative care.

# **Supplementary Table 3.** GRADE-CERQual evidence profile

| **Analytical theme** | **Contributing studies** | **Summary explanation** | **Methodological limitations** | **Coherence** | **Adequacy of data** | **Relevance** | **Overall confidence** | **Explanation of CERQual assessment** |
| --- | --- | --- | --- | --- | --- | --- | --- | --- |
| **Theme 1. Finding meaning through care for children and families** | 25 studies; 73 data units. Alm, 2026, Denmark; Bassola, 2023, Italy; Bloomer, 2015, Australia; Briere, 2025, USA; Brimble, 2024, UK; Budak, 2025, Turkey; Chong, 2017, Malaysia; Devitt, 2021, Ireland; Ebadinejad, 2022, Iran; Elsharkawy, 2025, Saudi Arabia; Epstein, 2008 and 2010, USA; Gürcan, 2025, Turkey; Henao-Castaño, 2019, Colombia; Hendricks-Ferguson, 2015, USA; Lewis, 2017, USA; McCloskey, 2010, UK; Özsavran, 2026, Turkey; Pant, 2026, UK; Papadatou, 1999, Greece; Ramadan, 2025, Egypt; Reid, 2013b, Scotland; Roach, 2023, Oman; Santos, 2014, Brazil; Stayer, 2016, USA; Suryani, 2018, Indonesia. | Through comfort care, communication, presence, and advocacy, nurses sought to provide care they regarded as worthwhile. Some described purpose, fulfillment, or professional value despite anticipated or actual loss. Frustration, helplessness, and limits on desired care also persisted. | Moderate concerns | Minor concerns | Minor concerns | Minor concerns | **Moderate** | Moderate methodological concerns remain, particularly incomplete positioning/reflexivity and congruity or reporting limitations in Özsavran (2026) and Papadatou (1999). Direct accounts of professional value strengthen the connection to meaning, but many contributing units describe care activities or barriers rather than personal meaning. Richness varies across meaning, advocacy, and wish-support. Respite/day care in Brimble (2024) and care across diagnosis/treatment in Roach (2023) add minor relevance concerns. Moderate confidence applies to the qualified finding about valued care and meaning, without assuming improved well-being or retention. |
| **Theme 2. Managing relational closeness and professional boundaries** | 27 studies; 63 data units. Abbasi, 2026, Iran; Alm, 2026, Denmark; Anguis-Carreño, 2023, Spain; Bassola, 2023, Italy; Bian, 2023, China; Bloomer, 2015, Australia; Briere, 2025, USA; Brimble, 2024, UK; Budak, 2025, Turkey; Chew, 2021, Singapore; Chong, 2017, Malaysia; Cook, 2012, USA; Devitt, 2021, Ireland; Elsharkawy, 2025, Saudi Arabia; Epstein, 2008 and 2010, USA; Erikson, 2017, USA; Gürcan, 2025, Turkey; Hendricks-Ferguson, 2015, USA; Jucker, 2024, Switzerland; Lewis, 2017, USA; McCloskey, 2010, UK; Pant, 2026, UK; Ramadan, 2025, Egypt; Reid, 2013a, Scotland; Reid, 2013b, Scotland; Shattnawi, 2022, Jordan; Stayer, 2016, USA. | Nurses managed closeness, emotional involvement, professional roles, confidentiality, and participation in care decisions. Some deliberately limited emotional involvement to continue working; others described numbness or depleted compassion. These experiences were distinct, and emotional availability was not uniformly maintained. | Moderate concerns | Minor concerns | Minor concerns | Minor concerns | **Moderate** | Methodological concerns remain moderate, including incomplete positioning/reflexivity and congruity/reporting concerns in Reid (2013a, 2013b). Deliberate distancing and involuntary emotional depletion are differentiated. Some units describe presence or attachment more directly than explicit boundary negotiation, and support is uneven across boundary facets. Some reports include wider care settings or professions. These combined concerns support Moderate confidence in the finding about managing closeness and professional roles. |
| **Theme 3. Responding to grief and loss through coping and meaning-making** | 29 studies; 76 data units. Abbasi, 2026, Iran; Anguis-Carreño, 2023, Spain; Bassola, 2023, Italy; Bian, 2023, China; Bloomer, 2015, Australia; Briere, 2025, USA; Budak, 2025, Turkey; Chew, 2021, Singapore; Chong, 2017, Malaysia; Cook, 2012, USA; Devitt, 2021, Ireland; Elsharkawy, 2025, Saudi Arabia; Erikson, 2017, USA; Gürcan, 2025, Turkey; Henao-Castaño, 2019, Colombia; Lewis, 2017, USA; Ma, 2021, China; MacDermott, 2014 & Keenan, 2016, Ireland; McCloskey, 2010, UK; Nugent, 2022, Ireland; Özsavran, 2026, Turkey; Pant, 2026, UK; Ramadan, 2025, Egypt; Reid, 2013a, Scotland; Reid, 2013b, Scotland; Santos, 2014, Brazil; Shattnawi, 2022, Jordan; Stayer, 2016, USA; Suryani, 2018, Indonesia. | Nurses experienced grief and emotional burden before, during, and after a child’s death. They responded through emotional expression, self-regulation, spiritual or cognitive meaning-making, avoidance, and self-care. Relief varied, and ongoing grief could coexist with coping efforts. | Moderate concerns | Minor concerns | Minor concerns | Minor concerns | **Moderate** | Moderate methodological concerns remain, including congruity/reporting issues in Özsavran (2026) and Reid (2013a, 2013b), and incomplete reflexivity. The finding includes persistent distress, delayed processing, and differing spiritual interpretations. The depth of evidence connecting specific coping responses with emotional regulation varies, and some self-care accounts are thin. Santos (2014) selected higher-resilience staff and includes unspecified nursing-professional categories. Moderate confidence concerns experiences and variable coping responses around loss, without assuming recovery. |
| **Theme 4. Seeking and using interpersonal and organizational support** | 30 studies; 90 data units. Abbasi, 2026, Iran; Anguis-Carreño, 2023, Spain; Bassola, 2023, Italy; Bian, 2023, China; Bloomer, 2015, Australia; Briere, 2025, USA; Brimble, 2024, UK; Budak, 2025, Turkey; Chew, 2021, Singapore; Chong, 2017, Malaysia; Cook, 2012, USA; Devitt, 2021, Ireland; Elsharkawy, 2025, Saudi Arabia; Henao-Castaño, 2019, Colombia; Hendricks-Ferguson, 2015, USA; Lewis, 2017, USA; Ma, 2021, China; MacDermott, 2014 & Keenan, 2016, Ireland; McCloskey, 2010, UK; Nugent, 2022, Ireland; Özsavran, 2026, Turkey; Pant, 2026, UK; Papadatou, 1999, Greece; Ramadan, 2025, Egypt; Reid, 2013b, Scotland; Roach, 2023, Oman; Santos, 2014, Brazil; Shattnawi, 2022, Jordan; Stayer, 2016, USA; Suryani, 2018, Indonesia. | Nurses sought and used peer, senior, personal, and organizational support while providing pediatric and neonatal palliative care. They valued understanding, guidance, learning opportunities, and resources. Access, uptake, and perceived usefulness varied; support gaps and experiences of unhelpful support were also reported. | Moderate concerns | No or very minor concerns | No or very minor concerns | Minor concerns | **High** | High confidence applies to the bounded finding on support needs, use, and variable usefulness. Coherence and adequacy raise no or very minor concerns: detailed accounts cover helpful, absent, inaccessible, and unhelpful support. Moderate methodological concerns, mainly incomplete reflexivity/positioning with additional reporting concerns in some studies, and minor relevance concerns remain. The core pattern also appears in studies with participant-supported findings and no identified analytic-congruity concerns (e.g., Budak (2025), Elsharkawy (2025), Pant (2026), Ramadan (2025)). Considered together, the limitations are judged unlikely to substantially alter this descriptive finding. High confidence does not establish support-program effectiveness or improved retention. |
